# Together is Better: mRNA Co‐Encapsulation in Lipoplexes is Required to Obtain Ratiometric Co‐Delivery and Protein Expression on the Single Cell Level

**DOI:** 10.1002/advs.202102072

**Published:** 2021-12-16

**Authors:** Heyang Zhang, Jeroen Bussmann, Florian H. Huhnke, Joke Devoldere, An‐Katrien Minnaert, Wim Jiskoot, Friedhelm Serwane, Joachim Spatz, Magnus Röding, Stefaan C. De Smedt, Kevin Braeckmans, Katrien Remaut

**Affiliations:** ^1^ Laboratory of General Biochemistry and Physical Pharmacy Faculty of Pharmaceutical Sciences Ghent University Ghent 9000 Belgium; ^2^ Division of BioTherapeutics Leiden Academic Center for Drug Research Leiden University Leiden 2333 CC The Netherlands; ^3^ Max Planck Institute for Medical Research Department of Cellular Biophysics 70569 Stuttgart Germany; ^4^ Department of Biophysical Chemistry University of Heidelberg 69120 Heidelberg Germany; ^5^ Center for NanoScience Ludwig‐Maximilian‐University Munich D‐80333 Munich Germany; ^6^ Faculty of Physics Ludwig‐Maximilian‐University D‐80539 Munich Germany; ^7^ Munich Cluster for Systems Neurology (SyNergy) D‐81377 Munich Germany; ^8^ RISE Research Institutes of Sweden Bioeconomy and Health, Agriculture and Food Göteborg 41276 Sweden; ^9^ Department of Mathematical Sciences Chalmers University of Technology and University of Gothenburg Göteborg 41296 Sweden; ^10^ Cancer Research Institute Ghent (CRIG) Ghent University Ghent 9000 Belgium; ^11^ Center for Advanced Light Microscopy Ghent University Ghent 9000 Belgium

**Keywords:** cellular uptake, mingle/single‐mRNA lipoplex, protein expression, single cell, theoretical modeling

## Abstract

Liposomes can efficiently deliver messenger RNA (mRNA) into cells. When mRNA cocktails encoding different proteins are needed, a considerable challenge is to efficiently deliver all mRNAs into the cytosol of each individual cell. In this work, two methods are explored to co‐deliver varying ratiometric doses of mRNA encoding red (R) or green (G) fluorescent proteins and it is found that packaging mRNAs into the same lipoplexes (mingle‐lipoplexes) is crucial to efficiently deliver multiple mRNA types into the cytosol of individual cells according to the pre‐defined ratio. A mixture of lipoplexes containing only one mRNA type (single‐lipoplexes), however, seem to follow the “first come – first serve” principle, resulting in a large variation of R/G uptake and expression levels for individual cells leading to ratiometric dosing only on the population level, but rarely on the single‐cell level. These experimental observations are quantitatively explained by a theoretical framework based on the stochasticity of mRNA uptake in cells and endosomal escape of mingle‐ and single‐lipoplexes, respectively. Furthermore, the findings are confirmed in 3D retinal organoids and zebrafish embryos, where mingle‐lipoplexes outperformed single‐lipoplexes to reliably bring both mRNA types into single cells. This benefits applications that require a strict control of protein expression in individual cells.

## Graphical abstract

Together is better: Red (R) and Green (G) fluorescent protein encoding mRNA encapsulated together in mingle‐lipoplexes (M_RG_1/1) result in the anticipated ratiometric co‐expression in each transfected cell in cell culture, retinal organoids and in vivo in zebrafish, while an unpredictable mixture of single‐green, single‐red and co‐transfected cells is obtained when a mixture of single‐green and single‐red lipoplexes (S_R_ S_G_1/1) is applied.

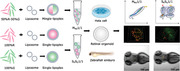

## 1 Introduction

In vitro transcribed messenger RNA (*IVT* mRNA) is a promising alternative to plasmid DNA (pDNA) to induce protein expression in a target cell, due to its ability to transfect non‐dividing cells, an earlier onset of protein expression, less risks of genome mutagenesis and the ease of scalable manufacturing procedures.^[^ ^1^ ^]^ Since the intrinsic instability of mRNA was improved by fine‐tuning the mRNA structure with modified nucleotides, cap analogues and optimized poly(A) tail lengths, the use of *IVT* mRNA has boomed for a variety of applications, including protein replacement, vaccination, treatment of cancer, gene editing and reprogramming.^[^ ^2^, ^3^ ^]^ Recently, for example, two mRNA vaccine candidates against the novel coronavirus (COVID‐19), i.e., BNT162b2 from Pfizer/BioNTech and mRNA‐1273 from Moderna, with over 94% efficacy and no serious safety concerns, have been authorized for emergency use in USA and Europe, leading to a worldwide recognition of mRNA as an emerging new drug class.^[^ ^4^, ^5^, ^6^ ^]^

To date, cationic lipids are the leading option for mRNA delivery which, when mixed with mRNA, spontaneously form nanoparticles that can enter cells through endocytosis while protecting mRNA against biodegradation in the extracellular and intracellular environments.^[^ ^7^, ^8^ ^]^ Furthermore, lipids can promote endosomal escape by destabilizing the endosomal membrane and inducing intracellular release of mRNA in the cytosol, where they can be translated into proteins.^[^ ^9^ ^]^ Despite the extensive use of lipid nanoparticles (LNPs) for mRNA delivery, fundamental questions remain on their structural organization. For example, it remains unclear how many mRNA strands are encapsulated per nanoparticle, with only very few studies having been carried out on this topic. Also, the amount of mRNA molecules that successfully escape from the endosomes after cellular internalization remains nothing more than an educated guess. Furthermore, several therapies require the simultaneous presence of a combination of RNAs, where it remains an open question as how to deliver all RNA types at the required stoichiometry into the cytosol of each individual cell. For genome editing, for example, co‐delivery of mRNA encoding the Cas9 nuclease and small guide RNA (sgRNA) has been widely evaluated to enhance the editing efficiency using different nanocarriers.^[^ ^10^ ^]^ Also the co‐delivery of a cocktail of 4 or more mRNAs encoding different reprogramming factors, is being used to generate human induced pluripotent stem cells.^[^ ^11^, ^12^ ^]^ Furthermore, co‐delivery of mRNAs encoding multiple proteins is of interest for vaccination as well.^[^ ^13^, ^14^ ^]^ For example, Lu et al. reported recently that a COVID‐19 mRNA vaccine delivering 3 mRNAs (encoding spike, membrane and envelope proteins) by LNPs induced a stronger immune response when compared to LNPs carrying spike protein mRNA alone.^[^ ^15^ ^]^ For all of these applications, multiple components must be co‐delivered in individual cells at an optimal ratiometric dose, which is quite challenging considering the stochasticity of the underlying processes, such as endocytic uptake and endosomal release. Robust co‐delivery methods, therefore, are of considerable importance to reliably control the delivery stoichiometry in individual cells both in vitro and in vivo.^[^ ^16^ ^]^

Given the fact that mRNA‐based nanomedicines are rapidly gaining access to clinics, we aimed to investigate the ability of liposomes to deliver a cocktail of two mRNAs in a pre‐defined ratio into single cells. To this end, we made use of two reporter mRNAs, encoding respectively enhanced green fluorescent protein (eGFP, green) and mCherry (red) which were complexed with a ‘gold‐standard‘ cationic lipid vector, Lipofectamine 2000, either separately (single‐lipoplexes) or together by pre‐mixing both mRNAs before encapsulation (mingle‐lipoplexes). For both single‐ and mingle‐lipoplexes, the average number of mRNA strands per lipoplex was quantified with Fluorescence Correlation Spectroscopy (FCS).^[^ ^17^, ^18^, ^19^ ^]^ Then, pre‐defined ratios of both mRNAs were applied onto cells either by mingle‐lipoplexes or a mix of single‐lipoplexes. The expression of both green and red fluorescent proteins was then quantified by flow cytometry and fluorescence microscopy at the single cell level. A theoretical framework was established to analyze and explain the experimental data, taking into account the stochasticity of lipoplex uptake and endosomal escape for both mingle‐ and single‐lipoplexes. Finally, the observed in vitro findings were confirmed in more complex 3D cellular structures (i.e., retinal organoids) and in vivo using zebrafish embryos. We found both experimentally and theoretically, that only mingle‐lipoplexes reliably brought both mRNAs at the desired ratio into single cells, while a mixture of single‐lipoplexes resulted in more variable protein expression. The fundamental findings in this study provide important insights in how to co‐deliver a cocktail of nucleic acids reliably at the required stoichiometry, which will benefit those applications where a strict control of protein expression on the individual cell level is required.

## 2 Results

### 2.1 Mingle‐Lipoplexes Containing Defined Ratios of Two mRNAs Produce Protein Expression at the Same Ratio on the Single Cell Level

First, to verify that it is sufficient to deliver mRNAs at a certain ratio on the single cell level in order to obtain expression of two proteins at that ratio, we delivered mixtures of unformulated mRNA encoding mCherry (red, R) or eGFP (green, G) into HeLa cells by nucleofection, which is an electroporation‐based transfection method that directly delivers mRNA into the cytosol of the cells.^[^ ^20^ ^]^ As seen in Figure S1 (Supporting Information), nucleofection results in successful co‐delivery of both mRNA types, where mCherry and eGFP protein expression is highly correlated (Figure S1E: Supporting Information, *R* ^2^ > 0.95), while the relative slopes of the best fit trend lines are proportional to the relative numbers of mCherry/eGFP mRNA that are delivered (Figure S1F, Supporting Information), even when the mean fluorescence intensity (MFI) values (Figure S1B, Supporting Information) show that eGFP is more easily detected and/or expressed as compared to mCherry.

**Figure 1:**
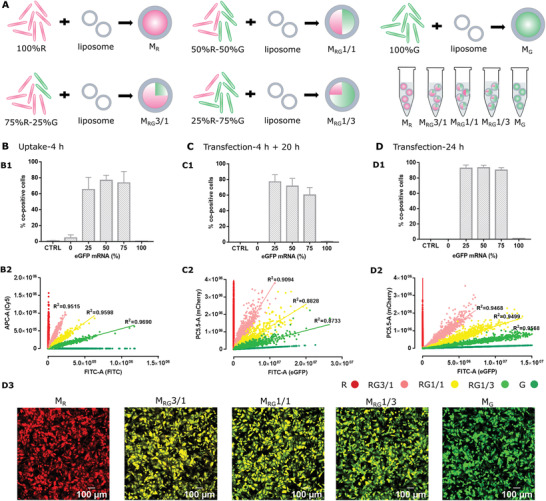
A) The mixture of Cy5 mRNA (red, R*) and FITC mRNA (green, G*) at an R*/G* ratio of 100/0, 75/25%, 50/50%, 25/75%, and 0/100% w/w was formulated into mingle‐lipoplexes, before being applied to cells. B) Mingle‐lipoplexes that contain Cy5 luciferase mRNA and FITC luciferase mRNA at an R*/G* ratio of 100/0%, 75/25%, 50/50%, 25/75%, and 0/100% w/w were incubated with HeLa cells for 4 h to examine the cellular uptake. C,D) Mingle‐lipoplexes that contain mCherry mRNA and eGFP mRNA at an R/G ratio of 100/0%, 75/25%, 50/50%, 25/75%, and 0/100% w/w were incubated with HeLa cells. C) After 4 h incubation, cells were rinsed and left to grow for another 20 h in fresh culture medium before flow cytometry measurements. D) After 24 h incubation (no washing step) cells were examined (D1, D2) by flow cytometry and (D3) by confocal microscopy. (B1, C1, D1) Percentage of co‐positive cells, (B2, C2, D2) correlation between red and green fluorescence as derived from flow cytometry dot plots. Scale bar: 100 µm. All the data was averaged from three independent experiments, with three replicates per repeat (*n* = 9). The total mRNA amount was kept constant at 0.2 µg/well.

Next, we prepared 5 different ratios of red (R*) and green (G*) fluorescently labeled mRNA by pre‐mixing them at these defined ratios and formulating them into mingle‐lipoplexes (M_R_, M_RG_3/1, M_RG_1/1, M_RG_1/3, and M_G_, respectively) (**Figure** **1**A). Using a previously published method for quantifying the number of mRNA strands per lipoplex using FCS^[^ ^17^ ^][^ ^21^ ^]^ we found that lipoplexes can accommodate on average 8 ± 2 mRNA strands per lipoplex. Assuming the pre‐mixed mRNA evenly distributes over all lipoplexes, this results in a ratio of Cy5 (R*)‐ to FITC (G*)‐labeled mRNA per lipoplex of 6/2, 4/4, and 2/6 for M_RG_3/1, M_RG_1/1, M_RG_1/3 respectively.

Importantly, incubating HeLa cells with mingle‐lipoplexes for 4 h resulted in cellular uptake of the pre‐defined R*/G* mRNA ratio on the single cell level. Indeed, the 5 different ratios can be clearly distinguished when plotting red versus green fluorescence as determined by flow cytometry and for each ratio the data are highly correlated (Figure 1, B2). Evidently no double‐labeled cells were detected for M_R_ or M_G_ for which only 1 type of mRNA was encapsulated, whereas the percentage of double‐labeled cells was consistently around 70% for M_RG_3/1, M_RG_1/1, and M_RG_1/3 lipoplexes containing both mRNA types (Figure 1, B1). Next, we checked how this translates to mCherry or eGFP expression by exposing cells to mingle‐lipoplexes with the same 5 ratios of mCherry‐ and eGFP‐mRNA (without fluorescent labels), followed by washing and an additional 20 h incubation to allow for protein expression. Again the 5 ratios could be clearly distinguished when plotting mCherry (R) versus eGFP (G) expression (Figure 1, C2) with the relative slopes of the best fit trend lines following the expected stoichiometry (Figure S3‐B4: Supporting Information). A good correlation was observed between mCherry and eGFP expression, even though it was slightly less as for particle uptake (Figure 1, C2). Of note, about 70% of cells expressed both mCherry and eGFP for the M_RG_3/1, M_RG_1/1, and M_RG_1/3 lipoplexes, whereas no co‐expressing cells were found for M_R_ and M_G_. The percentage of positive cells (Figure 1, D1) and the correlation of mCherry versus eGFP expression (Figure 1, D2) increased further when omitting the washing step at 4 h and letting the lipoplexes incubate for 24 h. It should be noted that also the MFI of the total population (∼delivery efficiency on the population level) follows accurately the applied R/G ratio (Figure S3‐A3, A4: Supporting Information). Taken together, these experiments demonstrate that mingle‐lipoplexes result in the expected R/G mRNA ratios both on a single cell level and as an average over the whole population.

### 2.2 A Ratiometric Mixture of Single‐Lipoplexes Containing One mRNA Type Rarely Produces Protein Expression at the Same Ratio on the Single Cell Level

In the next set of experiments, we formulated fluorescent R* or G* mRNA separately into lipoplexes, resulting in so‐called single‐lipoplexes (S) containing only one type of mRNA. Again, we calculated the number of mRNA strands per lipoplex by using FCS and found that both single‐R*‐ and single‐G*‐lipoplexes contain around 7 ± 1 mRNA strands per lipoplex. We assume this number will not change after mixing the single‐lipoplexes at 3/1, 1/1, and 1/3 R/G ratios, when mRNA strands are not exchanged between single‐lipoplexes in these mixtures.

**Figure 2:**
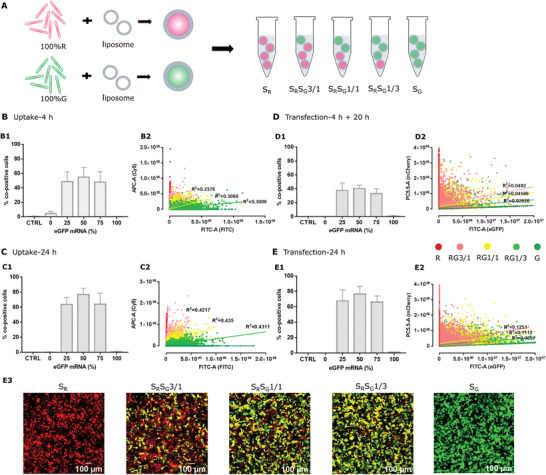
A) Cy5 mRNA or FITC mRNA is encapsulated separately into single‐lipoplexes, and the desired R*/G* ratios are obtained by mixing single‐R*‐ and single‐G*‐lipoplexes at an R*/G* ratio of 100/0%, 75/25%, 50/50%, 25/75%, and 0/100% w/w. B,C) Single‐lipoplexes mixtures that contain Cy5 luciferase mRNA single‐lipoplexes and FITC luciferase mRNA single‐lipoplexes at an R*/G* ratio of 100/0%, 75/25%, 50/50%, 25/75%, and 0/100% w/w were incubated with HeLa cells to examine the cellular uptake. B) After 4 h incubation, cells were rinsed and left to grow for another 20 h in fresh culture medium before flow cytometry measurements. C) After 24 h incubation (no washing step) cells were examined by flow cytometry. D,E) Single‐lipoplexes mixtures that contain mCherry mRNA single‐lipoplexes and eGFP mRNA single‐lipoplexes at an R/G ratio of 100/0%, 75/25%, 50/50%, 25/75%, and 0/100% were incubated with HeLa cells for (D) 4 h (followed by a washing step) or (E) 24 h (no washing step) and protein expression was determined at 24 h (E1, E2) by flow cytometry measurements and (E3) by confocal microscopy. (B1, C1, D1, E1) Percentage of co‐positive cells, (B2, C2, D2, E2) correlation between red and green fluorescence as derived from flow cytometry dot plots. Scale bar: 100 µm. All the data was averaged from three independent experiments, with three replicates per repeat (*n* = 9). The total mRNA amount was kept constant at 0.2 µg/well.

Again 5 different ratios of R/G mRNA were delivered to cells by incubating them with mixtures of single‐lipoplexes, namely S_R_, S_R_S_G_3/1, S_R_S_G_1/1, S_R_S_G_1/3, and S_G._ When the red or green fluorescence intensity obtained for uptake (B2, using labeled mRNA) and transfection (D2, using unlabeled mRNA) by single‐lipoplexes was examined at the individual cell level, significant differences appeared when compared with what we found for mingle‐lipoplexes (Figure 1, B2, C2). Indeed, cells are more randomly distributed in the R/G dot plots, leading to overlapping cell populations. Consequently, both the red and green fluorescence intensity levels after uptake (*R* ^2^ < 0.4) and transfection (*R* ^2^ < 0.05) are poorly correlated for the different R/G ratios that were applied onto the cells. The percentage of co‐expressing cells obtained with single‐lipoplexes is lower, being around 50% for cellular uptake (**Figure** **2**, B1), and 40% for eGFP and mCherry co‐expression (Figure 2, D1). Both the percentage of double‐labeled cells (Figure 2, C1, E1), as well as the correlation between red and green fluorescence improved by extending the exposure time of cells to single‐lipoplexes from 4 to 24 h (C2, *R* ^2^ ∼ 0.4; E2, *R* ^2^ ∼ 0.1), hence improving uptake and expression toward the expected ratios. However, the high correlation that was obtained by mingle‐lipoplexes already after 4 h (Figure 1, B2, *R* > 0.95) was never reached. In spite of this high variability on the single cell level, it should be noted that the cell population as a whole does exhibit the expected fluorescence ratios, as can be seen from the corresponding MFI values (Figure S4, A3, B3: Supporting Information).

**Figure 3:**
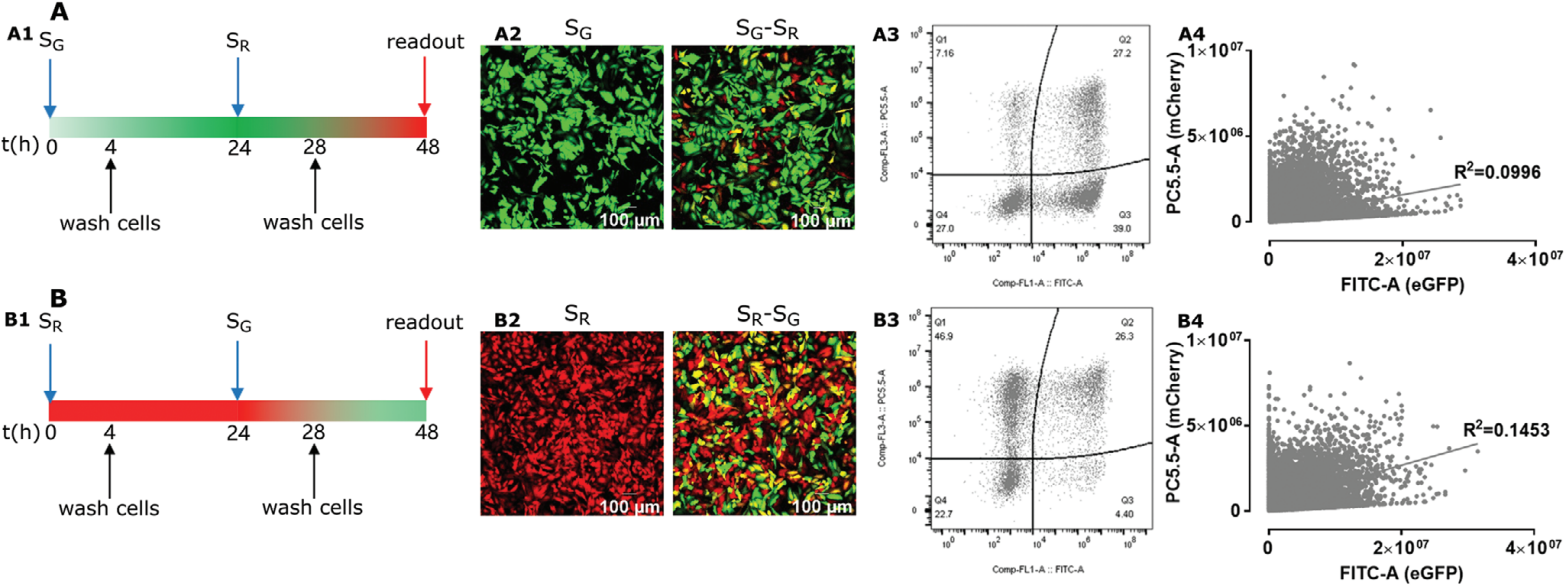
Subsequent transfection of eGFP mRNA single‐lipoplexes and mCherry mRNA single‐lipoplexes. A1,B1) Transfection scheme of cells, A2,B2) representative confocal images of cells after 1 time (left panel) and 2 times (right panel) transfection with single‐lipoplexes. A3,B3) Representative flow cytometry dot plots of cells receiving two subsequent transfections and A4,B4) correlation between red and green fluorescence as derived from flow cytometry dot plots. Scale bar: 100 µm. All the experiments have been triplicated in three independent days (*n* = 9). The mRNA dose was 0.1 µg/well per transfection round, leading to a total mRNA amount of 0.2 µg/well after the second transfection.

During simultaneous administration, competition might occur between S_R_ and S_G_ lipoplexes on the level of cellular entry, endosomal escape or cytosolic translation. Nevertheless, when cells were transfected first with S_G_ or S_R_ lipoplexes, followed by a subsequent transfection by an equal amount of S_R_ or S_G_ lipoplexes 24 h later, co‐delivery did not improve (**Figure** **3**). In contrast, cells seem even less likely to take up more lipoplexes and/or translate more proteins after being exposed to an initial lipoplex dose, resulting in only 25% of cells co‐expressing both G and R proteins, which is much lower than the 40% that was obtained when S_R_ and S_G_ lipoplexes were applied simultaneously. Instead, cells seem to favor expression of the protein corresponding to the first delivered mRNA (e.g., G for S_G_ + S_R_ and R for S_R_ + S_G_). Also, for 4 subsequent alternating transfections, these observations remained (Figure S5: Supporting Information).

In conclusion, single‐lipoplexes only result in the expected R/G mRNA ratios as an average over the whole population, whereas large single‐cell variations of the anticipated R/G ratios occur. This is in contrast to the mingle‐lipoplexes of which we found that they not only led to the expected R/G mRNA and protein ratios on the population level, but on the single cell level as well. As depicted in Figure S6 (Supporting Information), these important differences remain when performing multiple successive transfections in a row, where the correlation between mCherry and eGFP fluorescence on the single cell level remained consistently high for M_RG_1/1 lipoplexes (Figure S6C, Supporting Information), while it was again poorly correlated for S_R_S_G_1/1 lipoplexes after 1, 2, and 4 successive transfections (Figure 6D, Supporting Information).

### 2.3 Theoretical Modelling of Double mRNA Transfections by Mingle‐ and Single‐Lipoplexes

Experimentally we have observed that the correlation of mRNA uptake and protein expression by mingle‐ lipoplexes is very high, while this was not the case for single‐lipoplexes. At the same time, it was noticed that the correlation of mRNA uptake decreased for protein expression. It is of interest to see to which extent this can be theoretically understood, both qualitatively and quantitatively. As described in the (Part S2, Supporting Information), we derived a statistical model that describes cellular uptake and cytosolic release of cargo molecules (such as mRNA) delivered by single‐ and mingle‐lipoplexes. The model assumes a fixed number of *m* cargo molecules per lipoplex and considers the fractions *α*_1_ and *α*_2_ =  1 − *α*_1_ at which the two types of mRNA are included in a particular experiment. Note that for mingle‐lipoplexes, where both types of mRNA are incorporated into the same lipoplex formulation, the total number of mRNA molecules per lipoplex is still equal to *m*, but the relative numbers of both mRNAs can vary per lipoplex due to the stochastic nature by which mRNA molecules are incorporated into lipoplexes (following a binomial distribution, as explained in (Part S2, Supporting Information)). Following lipoplexes incubation with cells, the number of lipoplexes associated per cell will follow a certain distribution, denoted as *f_K_* (*k*). After association with cells, lipoplexes will have a finite probability *p_e_* to release their cargo into the cytosol (e.g., by direct fusion with the cell membrane or by endosomal release), leading to a certain distribution of the number of cytosolic cargo molecules per cell denoted as *f_X_* (*x*). Finally, the model assumes that the detected fluorescence in uptake experiments is proportional to the number of internalized mRNA molecules, or to the number of cytosolically released mRNA molecules in case of protein expression. The joint probability mass functions and the corresponding correlation functions were derived for the 4 relevant cases: 1) cargo uptake by single‐ and 2) mingle‐lipoplexes, 3) cytosolic cargo release and protein expression by single‐ and 4) mingle‐lipoplexes. As our focus is on understanding the changes in correlation between those cases, we here summarize the expressions for the correlation functions which can be derived without making any particular assumptions on the distributions *f_K_* (*k*) or *f_X_* (*x*):

1. Cell‐associated cargo delivered by single‐lipoplexes:
(1) $$\mathrm{Corr}\;\left(I_{1},I_{2}\right)=\frac{D\left(K\right)-1}{\sqrt{\left(1+\rho_{\alpha }D\left(K\right)\right)\left(1+D\left(K\right)/\rho_{\alpha }\right)}}\;$$
2. Protein expression after cytosolic cargo release by single‐lipoplexes:
(2) $$\mathrm{Corr}\;\left(I_{1},I_{2}\right)=\frac{D\left(X\right)-1}{\sqrt{\left(1+\rho_{\alpha }D\left(X\right)\right)\left(1+D\left(X\right)/\rho_{\alpha }\right)}}\;$$
3. Cell‐associated cargo delivered by mingle‐lipoplexes:
(3) $$\mathrm{Corr}\;\left(I_{1},I_{2}\right)=\frac{mD\left(K\right)-1}{\sqrt{\left(1+m\rho_{\alpha }D\left(K\right)\right)\left(1+mD\left(K\right)/\rho_{\alpha }\right)}}\;$$
4. Protein expression after cytosolic cargo release by mingle‐lipoplexes:
(4) $$\mathrm{Corr}\;\left(I_{1},I_{2}\right)=\frac{mD\left(X\right)-1}{\sqrt{\left(1+m\rho_{\alpha }D\left(X\right)\right)\left(1+mD\left(X\right)/\rho_{\alpha }\right)}}\;$$

where *I*_1_ and *I*_2_ refer to the observed fluorescence intensity (from labeled cargo or protein expression) from both types of cargo molecules, $\rho_{\alpha }=\frac{\alpha_{1}}{\alpha_{2}}\;=\frac{\alpha_{1}}{1-\alpha_{1}}\;$, $D\;\left(K\right)=\frac{\mathrm{Var}(K)}{E[K]}\;$and $D\;\left(X\right)=\frac{\mathrm{Var}(X)}{E[X]}\;$. D(*K*) and D(*X*) are the so‐called coefficients of dispersion of *f_K_* (*k*) and *f_X_* (*x*), respectively. It can be seen that the correlation functions have quite similar expressions for all 4 cases and only depend on the coefficients of dispersion of *f_K_* (*k*) and *f_X_* (*x*). In particular, the correlation functions are monotonically increasing functions of the coefficient of dispersion. Since *D*(*X*) ≤ *D*(*K*), as discussed in Part S2 (Supporting Information), this means that the correlation must decrease after endosomal escape, which corresponds to what we observed experimentally. Furthermore, since *m* ≥ 1 (i.e., there must be at least one mRNA molecule per lipoplex in order to form a lipoplex), we see that the correlation for mingle‐lipoplexes always must be larger than for the corresponding single‐lipoplex case, again in line with our experimental observations. A numeric example that visualizes these properties for the four correlation functions is shown in Figure S10 (Supporting Information) ( *α*_1_ = *α*_2_  =  0.5, *m*  =  5 and *p_e_* =  0.3). We also visualized the complete joint distributions for the case D (*K*) =  7 in Figure S11 (Supporting Information), where an over‐dispersed Poisson distribution was chosen to describe the lipoplex distribution over the cell population.^[^ ^22^ ^]^ This example visually shows how the correlation is indeed improved when delivery happens by mingle‐ instead of single‐lipoplexes (compare Figure S11, B with A and D with C, Supporting Information). Also the loss in correlation upon cargo release and protein expression is apparent (compare Figure S11, C with A and D with B, Supporting Information) since the distributions become less elongated, or more spherical so to say. The data for mingle‐ and single‐lipoplexes also indicated that the correlation of protein expression improves when prolonging the exposure to lipoplexes from 4 to 24 h. This is predicted by the model as well since evidently the chance for cytosolic cargo release is larger over a period of 24 h instead of 4 h. Indeed, as *p_e_* increases so will D(*X*), leading to an increase in correlation considering the monotonicity of the correlation functions with D(*X*).

**Figure 4:**
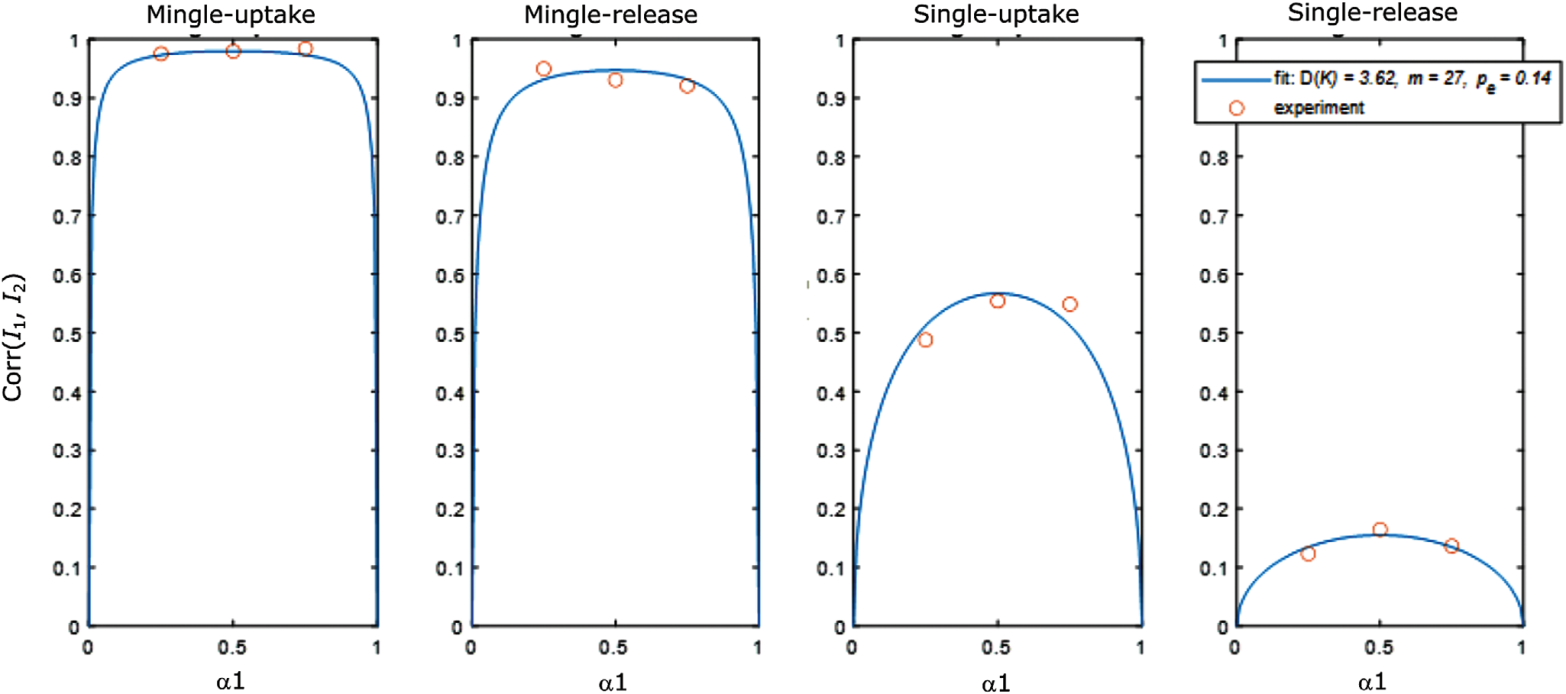
Simultaneous best fit (blue solid line) of the 4 correlation functions to the experimental correlation values (red circles) with D(*K*), *p_e_* and *m* as global fitting parameters. From left to right the four considered conditions are displayed in order of decreasing correlation: cargo uptake delivered by mingle‐lipoplexes, cytosolically released cargo from mingle‐lipoplexes, cargo uptake by single‐lipoplexes and cytosolically released cargo from single‐lipoplexes. Per condition the three considered cargo amounts are included: *α*_1_ =  0.25, 0.5, and 0.75 (corresponding to *α*_1_ =  0.75, 0.5, and 0.25, respectively).

Apart from this qualitative agreement between theory and experiments, it is of interest to see if the model quantitatively agrees with the measured correlation values. As discussed in the SI (Part 2) this can be done by fitting all four correlation functions simultaneously to the experimental correlation values with D(*K*), *p_e_* and *m* as global fitting parameters. When we do a least squares fit of the model to the experiments where cells were incubated with the lipoplexes for 4 h, we indeed find that it nicely describes the experimental correlation data for D (*K*) =  3.62, *p_e_* =  0.14 and *m*  =  27.0 (**Figure** **4**). Also for these fitting parameters, the simulated histograms showed a clear increase in correlation when mingle‐lipoplexes are used instead of single‐lipoplexes, both for uptake (Figure S12A vs S12B: Supporting Information) and expression (Figure S12C vs S12D: Supporting Information), which is nicely in line with what was experimentally observed (Figure 1B2 vs Figure 2B2 and Figure 1C2 vs Figure 2D2). In addition, there is a loss in correlation when going from uptake to expression, both for single‐ (Figure S12A vs 12C: Supporting Information) and mingle‐lipoplexes (Figure S12B vs S12D: Supporting Information), again corresponding to what was observed experimentally (Figure 1B2 vs Figure 1C2 and Figure 2B2 vs Figure 2D2). Finally, also visually one can appreciate the similarity in the ‘shapes’ of the various theoretical distributions as compared to the experimental ones. For lipoplexes that are exclusively internalized by endocytosis, *p_e_* gives an indication of the endosomal escape probability, which is quite interesting as it is very difficult to determine by any other means. Considering that cargo release is one of the major bottle necks for nanomedicines at the intracellular level, a release probability of 14% seems plausible, even though more experiments would be needed to verify this number. The model furthermore suggests that 27 mRNA molecules must have been incorporated per lipoplex on average, which is more than the 8 ± 2 mRNA molecules that was determined by FCS. The discrepancy between them remains unclear at the moment, especially since there are no other established techniques to measure this quantity. Nevertheless, we find that three global parameters are sufficient to find a quantitative agreement between the derived model and experimental correlation data for all considered conditions, being a strong indication that the model does capture the most essential processes that lead to the observed results.

### 2.4 Mingle‐Lipoplexes Result in More Precise Co‐Transfection of Cells in Ex Vivo Retinal Organoids and In Vivo in Zebrafish Embryos

**Figure 5:**
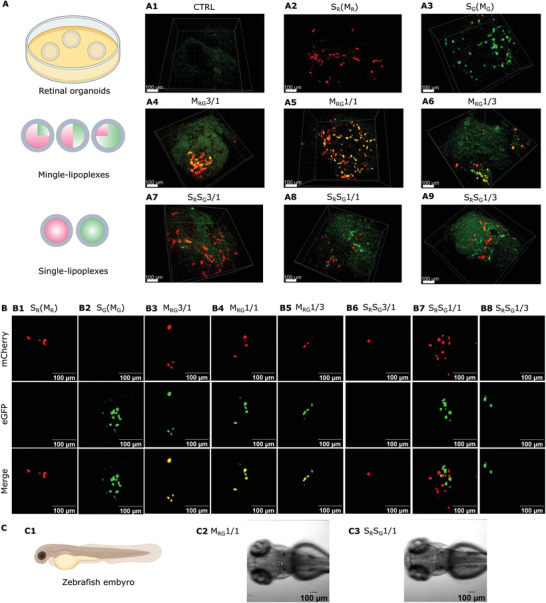
Representative images of A) retinal organoids and B,C) zebrafish embryos transfected with mRNA lipoplexes. (A) Briefly, retinal organoids at day 22 of cultivation were incubated with (A2) 100% mCherry mRNA lipoplexes, (A3) 100% eGFP mRNA lipoplexes, (A4‐A6) mingle‐lipoplexes containing mCherry/eGFP mRNA at a ratio of 75/25%, 50/50%, 25/75% w/w or (A7‐A9) mixtures of mCherry mRNA single‐lipoplexes and eGFP mRNA single‐lipoplexes at a ratio of 75/25%, 50/50%, and 25/75% w/w. The non‐treated retinal organoids were used as control (A1). All the experiments were performed in triplicate (*n* = 9). At 24 h post‐administration, retinal organoids were fixed by 2% PFA for images by microscopy. Scale bar: 100 µm. B) Zebrafish embryos of 48 h post fertilization were microinjected at the hindbrain ventricle with (B1) 100% mCherry mRNA lipoplexes, (B2) 100% eGFP mRNA lipoplexes, (B3‐B5) mingle‐lipoplexes containing mCherry/eGFP mRNA at a ratio of 75/25%, 50/50%, 25/75% w/w or (B6‐B8,) mixtures of mCherry mRNA single‐lipoplexes and eGFP mRNA single‐lipoplexes at a ratio of 75/25%, 50/50%, and 25/75% w/w. At 24 h after injection, embryos were embedded in agarose and imaged using confocal microscope at 40x magnification (72 h post fertilization in total). C) Representative images of zebrafish embryos 24 h after injection (72 h post fertilization) at 10x magnification. (C1) schematic of a zebrafish embryo, (C2) mingle‐lipoplexes containing mCherry/eGFP mRNA at a ratio of 50/50% (w/w and (C3) mixtures of mCherry mRNA single‐lipoplexes and eGFP mRNA single‐lipoplexes at a ratio of 50/50% w/w). Around 15–20 zebrafish embryos were injected for each sample. Scale bar: 100 µm. The total mRNA amount was kept constant at 1 µg/well for retinal organoids, and 0.2 ng/injection for zebrafish embryos.

To confirm our in vitro findings, we performed the co‐delivery of mCherry and eGFP mRNA by mingle‐ or single‐lipoplexes in more complex cellular systems, namely mouse embryonic stem cell‐derived 3D retinal organoids and zebrafish embryos. As **Figure** **5** shows, both mCherry and eGFP mRNA can be respectively translated into mCherry proteins and eGFP in the organoids transfected with single‐R‐ or single‐G‐lipoplexes for 24 h (A2, A3), in comparison with the negative control (A1). When mixtures of mCherry and eGFP mRNA were administered by M_RG_3/1, M_RG_1/1, and M_RG_1/3, each transfected cell shows the co‐expression of both R and G proteins (A4‐A6), whereas co‐expression of R and G proteins in individual cells of the organoids transfected with a mixture of S_R_S_G_3/1, S_R_S_G_1/1, or S_R_S_G_1/3 lipoplexes (A7‐A9) was very rare. Instead, most cells display only G or only R fluorescence, demonstrating that the chance that both an S_G_ and an S_R_ complex co‐transfected the same cell is very limited.

Given the limited possible injection volume of 2 nL (containing 0.2 ng mRNA) in zebrafish as an in vivo model, we first confirmed that microinjection of this low amount of mRNA (free mCherry, free eGFP or a 50/50 mixture of both) into zebrafish embryos at the 1‐cell stage resulted in detectable expression of both types of fluorescent proteins (Figure S7, Supporting Information). Then, 2 nL of lipoplexes containing 0.2 ng mRNA were injected into the hindbrain ventricle of zebrafish embryos at 48 h post fertilization (hpf) based on a previous report.^[^ ^23^ ^]^ At 24 h post‐injection of mingle‐ or single‐lipoplexes, in general around 30–50% of embryos showed detectable fluorescence protein expression, confined within a small number of cells in the brain. Additionally, we observed a good survival (100%) of embryos 24 h after injection (i.e., 72 hpf) with both mingle‐ and single‐lipoplexes. Importantly, embryos injected with M_RG_3/1, M_RG_1/1 or M_RG_1/3 lipoplexes showed co‐expression of both R and G proteins in each transfected cell (Figure 5, B3‐B5, C2), whereas injection with S_R_S_G_3/1 and S_R_S_G_1/3 lipoplexes resulted in a very few positive cells expressing either R or G proteins respectively (Figure 5, B6, B8). Furthermore, even though S_R_S_G_1/1 lipoplexes resulted in both R and G expressing cells, the co‐expression of R and G proteins in a single cell was never found (Figure 5, B7, C3). This again confirms that to reliably deliver two mRNA types, co‐encapsulation into lipoplexes is required to ensure simultaneous cellular uptake, endosomal escape and translation of both mRNA types on the individual cell level.

## 3 Discussion

Co‐administration of multiple mRNA types is of interest for any application requiring the expression of more than one type of protein, such as multi‐target vaccination strategies and the expression of protein cocktails for regeneration purposes. In this work, we demonstrated that in order to obtain reliable ratiometric dosing on the single‐cell level, all mRNA types must be encapsulated into the same nanoparticle (lipoplexes in our case). The distinct ability of mingle‐lipoplexes to deliver their mRNA mixture into individual cells was even more notable in more hard‐to‐transfect conditions, like 3D cultured retinal organoids and zebrafish embryos.

A prerequisite to use mingle‐complexes for co‐delivery, is that different mRNA types can “fit” into the same nanoparticles. In‐depth characterization on the number of mRNA molecules per complex are however scarce due to the limited available methods and techniques. Also, the mRNA size, the carrier type, the chosen carrier to mRNA ratio and the final nanoparticle size will influence the number of mRNA strands present per complex. In this work, with FCS we determined that lipoplexes contain on average 8 ± 2 mRNA strands. Sabnis et al. experimentally determined that on average 5 mCherry mRNA strands (≈996 nt) per lipoplex of 80 nm were present.^[^ ^24^ ^]^ In a very recent study, Carrasco et al. determined 6 mRNA for LNP particles of about 65 nm diameter.^[^ ^25^ ^]^ Arteta et al. found 5 mRNA (858 nt) for 50 nm LNPs and 200 mRNA for 140 nm LNPs based on volume fraction arguments.^[^ ^26^ ^]^ Finally, Leonhardt et al. reported on average 350 mRNA molecules (1192 nt) per lipoplex of LF2000/mRNA at a v/w ratio of 2.5, by measuring the size and packing density of lipoplexes.^[^ ^27^ ^]^ As the quantification methods and mRNA nanoparticle formulations are different, the reported number of mRNA molecules per nanoparticle is quite variable. Also, the number of mRNA per nanoparticle depends strongly on size. When applying our here derived statistical model to the experimental correlation values, an average of 27 mRNA molecules per lipoplex was estimated, which was higher than our experimental findings with FCS but within the range as reported by Arteta et al.^[^ ^26^ ^]^ In any case, it is quite remarkable that a plausible number of mRNA molecules per lipoplex follows from the analysis of uptake and expression correlation values. Recently, Gómez‐Varela et al. used cross‐correlation analysis to determine the association of red labeled plasmid DNA molecules to green labeled liposomes and revealed about 6 pDNA molecules per lipoplex.^[^ ^28^ ^]^ A similar cross‐correlation approach could be useful to investigate mRNA association to LNPs and lipoplexes, to further in depth characterize the influence of lipid nanoparticle composition and size.

**Figure 6:**
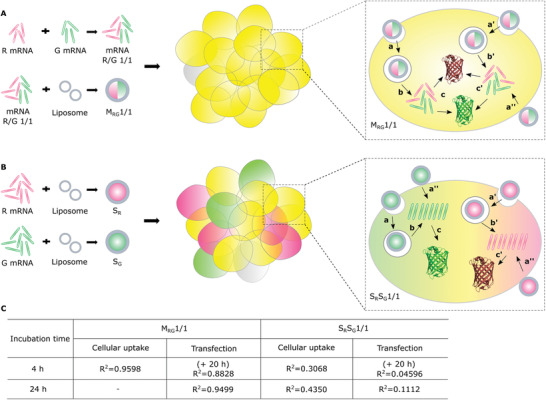
Schematic illustration of A) mRNA mingle‐lipoplexes and B) mRNA single‐lipoplexes at an R/G ratio of 1/1 that are being applied onto cells. (a, a’) mRNA lipoplexes are internalized into cells through endocytosis or (a’’) cell membrane fusion, (b, b’) the internalized lipoplexes escape from endosomal compartments, releasing free mRNA into the cytosol of cells, (c, c’) cytosolic mRNA can be translated leading to the target protein expression. C) Correlation derived from the flow cytometry dot plots of HeLa cells that were respectively applied with M_RG_1/1 and S_R_S_G_1/1. For cellular uptake, mRNA lipoplexes were incubated with cells for 4 and 24 h, while for transfection, mRNA‐lipoplexes were incubated with cells for 4 h + 20 h and 24 h, respectively.

Clearly, mingle‐lipoplexes outperformed a mixture of single‐lipoplexes to deliver multiple mRNA types into the cytosol of individual cells according to pre‐defined R/G mRNA ratios. **Figure** **6** summarizes the intracellular journey of mingle‐ and single‐lipoplexes with a representative total R/G mRNA ratio of 1/1. Lipoplexes attachment to cells, cell uptake and cytosolic cargo release are all stochastic processes. Assuming that each mingle‐lipoplex contains 4 R‐ and 4 G‐mRNA molecules, this brings them simultaneously into the cell when a lipoplex is endocytosed or fuses with the plasma membrane (Figure 6A, step a, a’, a’’), leading to a high correlation (*R* ^2^ > 0.95 at 4 h uptake). For single‐lipoplexes, however, half the amount contains 8 R‐mRNA molecules, while the other half contains 8 G‐mRNA molecules. The precise number of single‐lipoplexes of type 1 and 2 that releases their cargo in the cytosol of a particular cell may be quite variable, leading to a rather poor correlation on the single cell level (*R* ^2^ < 0.31 at 4 h uptake). Increasing the exposure time of lipoplexes to cells from 4 to 24 h improved the correlation to some extent (to *R* ^2^ < 0.44), as more lipoplexes will be taken up and release their cargo, but never equaled those obtained for the mingle‐lipoplexes already after 4 h. Also performing subsequent transfections, to rule out a possible competition between S_G_ and S_R_ lipoplexes does not improve correlation (Figure 3 and Figure S5: Supporting Information). When performing an uptake or transfection experiment, the transfection medium contains 4.25 × 10^10^ lipoplexes for 10^5^ cells, being 425 000 lipoplexes/cell. When each cell would internalize hundreds of lipoplexes, it would be statistically expected that the extracellular 1/1 ratio would be gradually approached. This readily follows from the derived statistical model as well. As discussed in the (Part S2, Supporting Information), the number of cell‐associated lipoplexes can be modelled by a negative binomial distribution *f_k_* (*k*) =  NegBino(*k*; *κ*, *θ*) with coefficient of dispersion *D* (*K*) =  1 + *θ*. As elaborated upon in the SI, if the average number of lipoplexes per cell E[*K*] increases (e.g., by adding a higher concentration of lipoplexes or by increasing the incubation time) the parameter *θ* would also increase; hence, *D*(*K*) and therefore the correlation would also increase. As Leonhardt et al. showed, however, the number of lipoplexes endocytosed into cells is rather low (between 4–15 lipoplexes per cell), contributing to the poor correlation observed for single‐lipoplexes.^[^ ^27^ ^]^ Our results thus seem to indicate that the uptake and expression results are rather determined by the individual composition of mRNA molecules per lipoplex, rather than the overall composition that is applied onto cells.

Lipoplexes need to fuse with the endosomal membrane (endosomal escape, Figure 6, step b, b’) or with the cell membrane (Figure 6, step a’’) to deliver their mRNA payloads into the cytosol of the cells, after which mRNA can be translated into proteins (Figure 6, step c, c’).^[^ ^29^ ^]^ An interesting fact of our statistical model is that it gives an indication of the probability *p_e_* that a lipoplex successfully releases its cargo into the cytosol. By fitting of the model to the experimental correlation values, we obtain a cytosolic release value *p_e_* =  14%, which seems quite plausible compared to endosomal escape efficiencies between 2.5% and 30% reported by Sabnis et al. and Patel et al., using a single‐molecule fluorescence microscopy‐based assay.^[^ ^24^ ^][^ ^30^ ^]^ Further validation of this model could thus provide an extra tool to estimate endosomal escape efficiency, which is relatively easy compared to the other assays and techniques that are currently available.^[^ ^29^, ^31^, ^32^ ^]^ It should be noted that the fitted parameters *D*(*K*) and *p*_e_ depend on the concentration of lipoplexes that was applied and/or the time at which the data is recorded after adding the lipoplexes to the cells. Indeed, the concentration of lipoplexes and incubation time can influence the average number and variance of lipoplexes per cell, and therefore $D\;\left(K\right)=\frac{\mathrm{Var}(K)}{E[K]}\;$as well. In addition, *p*_e_ can be time‐dependent as endosomal escape efficiency may require endosomal maturation to occur, which is a time‐dependent process. The rather low cytosolic release efficiency further contributes to the fact that the chance for an individual cell to first successfully internalize single‐lipoplexes and then translate proteins of the escaped mRNA at the desired R/G ratio is rather limited. In more complex cellular structures, like 3D retinal organoids, where cells are more densely packed and cellular uptake is even more challenging, this even resulted in the absence of cells that simultaneously expressed red and green fluorescent proteins when single‐lipoplexes were used (Figure 5). In addition, in zebrafish embryos transfected with S_R_S_G_ lipoplexes at a 3/1 and 1/3 ratio, the low probability of cellular uptake and endosomal escape resulted in the absence of detectable protein expression for the lowest fraction of S_G_ and S_R_ lipoplexes, respectively. On the other hand, mingle‐lipoplexes always succeeded in bringing both the R and G mRNA they carry into the same cell, even in hard‐to‐transfect cells in ex vivo and in vivo conditions.

A final important observation was that transfection with single‐lipoplexes seems to follow the “first come, first serve” principle, where the chance of transfecting an “already transfected” cell is drastically lowered when compared to the transfection of a naive cell population (Figure 3). A possible factor contributing to this observation is the innate immune system that is triggered upon mRNA recognition by toll like receptors (TLRs) in the endosomal compartment and that hampers the translation of the delivered mRNA molecules.^[^ ^33^ ^]^ Also mingle‐lipoplexes can trigger this innate immune system, although each successful uptake and endosomal escape event will simultaneously deliver both R and G mRNA that can be translated as long as the innate immunity threshold for mRNA degradation is not reached. Therefore, while the level of protein expression can differ from low to high from cell to cell (depending on the amount of successful delivery events in the immune‐silent time frame), the ratio of R to G protein expression is always strongly correlated (when R is high, also G is high and vice versa). For single‐lipoplexes, however, both the uptake and endosomal escape of R and G mRNA happen independently, leading to a high variety of intracellular mRNA mixtures that can reach the cytosol in the immune‐silent window, and resulting in a poor correlation of intracellular protein expression levels, regardless of the R/G ratio that was applied onto the cells. This might also explain why when performing multiple transfections in a row, the number of cells co‐expressing both R and G mRNA seemed to gradually decrease, while intuitively, one might expect the percentage of co‐expressing cells to increase (Figure S6: Supporting Information).

Although this study focused on the delivery of two mRNA types, the co‐delivery of different nucleic acid types, like siRNA, pDNA and oligonucleotides is of interest as well.^[^ ^34^ ^]^ Schwake et al., for example studied co‐delivery of two plasmids and demonstrated that both plasmids are simultaneously expressed, suggesting that they are delivered in correlated units (e.g., by the same complex).^[^ ^35^ ^]^For genome editing, for example, co‐delivery of mRNA encoding the Cas9 nuclease and sgRNA has been widely studied to enhance the editing efficiency using different nanocarriers.^[^ ^36^, ^37^ ^]^ Also the co‐delivery of mRNA with short interfering RNA (siRNA) is of interest to introduce therapeutic protein expression, while simultaneously downregulating the disease causing protein.^[^ ^38^ ^]^ Other combinations of same or different nucleic acid types, such as siRNA/pDNA, miRNA/siRNA, pDNA/pDNA, have been explored.^[^ ^39^, ^40^, ^41^ ^]^ Rehman et al delivered an 1:1 ratio of two ODNs and found that most cells were positive for both ODNs when co‐delivered by PEI polyplexes or Lipofectamine2000 lipoplexes, while respectively up to 80% and 20% of cells were only positive for either one of the ODNs when two separate polyplexes or lipoplexes were used, demonstrating that the importance of co‐encapsulation is also carrier dependent.^[^ ^42^ ^]^ Also the co‐delivery of pDNA and oligonucleotides is of use as an endosomal escape assay^[^ ^42^ ^]^.^[^ ^43^ ^]^ Of note, the preparation of mingle‐complexes for these applications will only be beneficial when the type of chosen nanoparticle (e.g., lipid or polymer composition) suits all nucleic acid types to be delivered. Whenever this one‐fits‐all requirement is not fulfilled, single‐complexes offer more flexibility to tailor the nanoparticle composition to the individual nucleic acid delivery requirements. The risk that not all nucleic acids reach the same individual cell, however, sharply increases in the case of single‐lipoplexes, especially when the number of complexes that successfully travels from the place of administration to the eventual target cell can be very low in vivo. When ratiometric dosing should only be achieved on the population level, however, also mixtures of single‐lipoplexes could still be used.

## 4 Conclusion

In this report, we investigated mRNA mingle‐lipoplexes and single‐lipoplexes as well as their biological performance in cancer cells, retinal organoids and zebrafish embryos. We found that each lipoplex contains on average 8 mRNAs per lipoplex, with a composition that equals the mRNA mixture before encapsulation. Furthermore, mingle‐lipoplexes resulted in a nice correlation between red (Cy5 or mCherry) and green (FITC or eGFP) fluorescence intensity on the single cell level, which was consistent to the initial R/G mRNA ratio, both in vitro, in retinal organoids and zebrafish embryos. In contrast, single‐lipoplexes only resulted in the delivery of the anticipated mRNA ratio on average over the whole population, albeit with highly variable and poorly correlated expression ratios on the single‐cell level. Furthermore, in hard‐to‐transfect cellular conditions like retinal organoids and zebrafish, successful co‐delivery using single‐lipoplexes was rarely observed. These observations were confirmed by a statistical model that describes mRNA complexation, cellular uptake and cytosolic release of cargo molecules (such as mRNA) delivered by single‐ and mingle‐lipoplexes. To conclude, mRNA molecules should be encapsulated into the same nanoparticles to reliable bring them to the cytosol of individual cells, providing the number of mRNAs to be delivered does not exceed the encapsulation capacity of the delivery vector.

## 5 Experimental Section

### General

5‐Methoxyuridine (5moU) modified messenger RNA encoding enhanced Green Fluorescence Protein (eGFP mRNA), mCherry (mCherry mRNA) and luciferase protein (Luc mRNA) were purchased from Trilink BioTechnologies (San Diego, CA). Lipofectamine 2000 (LF2000), Opti‐MEM, Dulbecco's Modified Eagle's Medium/F12 (DMEM/F12), 0.25% trypsin/EDTA, L‐glutamine, penicillin‐streptomycin (5000 U mL^−1^) and DPBS[‐] were purchased from Thermo Fisher Scientific (Merelbeke, Belgium). HEPES was purchased from Sigma‐Aldrich (Overijse, Belgium). Fluorescent labeling of mRNA by Cy5 or Fluorescein (FITC) was performed by using the Label‐IT nucleic acid labeling kit (Mirus Bio, Madison, WT, USA). For FCS measurements, eGFP mRNA and mCherry mRNA were labeled by Cy5 and FITC respectively, while for uptake measurements, luciferase mRNA was fluorescently labeled by Cy5 or FITC, according to the protocol provided by the manufacturer.

### mRNA Lipoplexes Preparation

Mingle. For mingle mCherry/eGFP‐containing lipoplexes, mRNA mixtures containing 100/0%, 75/25%, 50/50%, 25/75%, and 0/100% w/w of mCherry mRNA and eGFP mRNA were prepared by mixing the corresponding volume ratios of mRNA stock solutions (0.1 µg µL^−1^). For clarity, these will be abbreviated to M_R_, M_RG_3/1, M_RG_1/1, M_RG_1/3 and M_G_, respectively, throughout the manuscript. For FCS or uptake measurements, mRNA mixtures were prepared by FITC‐labeled mRNA (green, G* as a model for eGFP mRNA) and Cy5‐labeled mRNA (red, R* as a model for mCherry mRNA). After premixing the appropriate mRNAs, the mRNA mixture was formulated into mingle‐lipoplexes by LF 2000 at v/w ratio of 3:1 (µL µg^−1^) according to the protocol provided by the manufacturer. The resulting lipoplexes were used as such, without further washing or dialysis.

Single. For single mCherry or eGFP‐containing lipoplexes, mCherry mRNA or eGFP mRNA was formulated with LF2000 at v/w ratio of 3:1 (µL µg^−1^), according to the protocol from the manufacturer. The resulting lipoplexes were used as such, without further washing or dialysis. For FCS or uptake measurements, FITC‐labeled mRNA (green, G* as a model for eGFP mRNA) or Cy5‐labeled mRNA (red, R* as a model for mCherry mRNA) was separately formulated in the single‐lipoplexes. Then, mixtures containing 100/0%, 75/25%, 50/50%, 25/75%, and 0/100% w/w mCherry mRNA (or Cy5 mRNA) and eGFP mRNA (or FITC mRNA) were prepared by mixing the corresponding volume ratios of both types of mRNA single‐lipoplexes. For clarity, these will be abbreviated to S_R_, S_R_S_G_3/1, S_R_S_G_1/1, S_R_S_G_1/3 and S_G_ throughout the manuscript. Please note that when only one type of mRNA is delivered, M_R_ and S_R_ or M_G_ and S_G_ are basically the same.

### Size and Zeta Potential Characterization of mRNA Lipoplexes

The average size and *ζ* potential of lipoplexes was measured by using a Zetasizer Nano‐ZS (Malvern, Worcestershire, UK) in HEPES buffer. Lipoplexes were diluted with 25 × 10^−3^ m HEPES buffer (pH7.4) to a final concentration of 0.2 µg mL^−1^ eGFP or mChery mRNA. The size and *ζ* potential of mingle‐lipoplexes (M_RG_1/1), single‐G‐lipoplexes and single‐R‐lipoplexes was 374 ± 47 nm and −16 ± 3 mV, 310 ± 57 nm and −20 ± 3 mV and 323 ± 30 nm and −18 ± 2 mV respectively.

### Quantification of mRNA per Complex by Using FCS

Fluorescent lipoplexes containing FITC‐labeled mRNA (green) as a model for eGFP mRNA (996 nt) and Cy5‐labeled mRNA (red) as a model for mCherry mRNA (996 nt) were prepared by both the mingle and single method as described above. 50 µL of sample was loaded on a glass‐bottomed 96‐well plate (Grainer Bio‐one, Frickenhausen, Germany) for the FCS measurements. The detection volume of the FCS instrument was calibrated by Alexa 647 (*V*_eff_ of 0.35 ± 0.02 fL) and Rhodamine green (*V*_eff_ of 0.55 ± 0.03 fL) respectively. Fluorescence time traces (60 s) were obtained by focusing a 640 and 488 nm laser line for Cy5 mRNA and FITC mRNA, respectively through a water immersion objective lens (60x Plan Apo VC, N.A. 1.2, Nikon, Japan) at about 50 µm above the bottom of the plate. The green and red fluorescence signal was recorded by a photon counting instrument (PicoHarp 300, PicoQuant). The complexation efficiency or association degree (%) of mRNA to lipoplexes was directly determined from these fluorescent fluctuations as described before^[^ ^17^ ^][^ ^21^ ^]^ and was 93.4 ± 2.2% for S_R_ or M_R_ and 91.5 ± 4.8% for S_G_ or M_G_ respectively.

To determine the number of mRNA molecules per complex, the fluorescence fluctuation profiles were further analyzed by fitting the resulting autocorrelation curves by a single‐species or dual‐species model using SymPhoTime software. With auto‐correlation analysis, first, the concentration of free green or red fluorescent mRNA molecules before complexation was measured by using FCS (single‐species fit, C_mRNA_). Then, mRNA was encapsulated into lipoplexes and FCS was again used to determine the concentration of (green or red fluorescent) lipoplexes that were formed (dual‐species fit, C_LPX_). The number of nucleic acids per lipoplex was determined by dividing the concentration of the initial free mRNA by the concentration of resulted lipoplexes, taking into account the association degree as determined above (C_mRNA_*(association degree/100)/ C_LPX_) (Figure S2: Supporting Information).

### In Vitro Cellular Uptake and Transfection

Cell culture. HeLa cells (ATCC, Manassas, USA) were cultivated in DMEM/F12 with 10% Fetal Bovine Serum (FBS), 2% penicillin‐streptomycin and 1% L‐glutamine at 37°C, 5% CO_2_. Cells with 80–90% confluency were detached from the bottom of the flask with 0.25% trypsin/EDTA. For all in vitro experiments (apart from the retinal organoids), cells were seeded in 24‐well plates (50 000 cells/well) at 37°C, 5% CO_2_ one day before treatment.

Nucleofection. By using a 4D Nucleofector (Lonza, Bioscience, Bornem, Belgium), 2 µL of mRNA mixtures (i.e., 1.5 µg/0.5 µg, 1.0 µg/1.0 µg, and 0.5 µg/1.5 µg of mCherry/eGFP mRNA) was added to 18 µL of cell suspension (containing 2.6 × 10^5^ cells) in each cell strip for nucleofection. After 24 h incubation at 37°C, the cells were harvested for the measurements by flow cytometry (Cytoflex, Beckman Counter, Suarlée, Belgium).

Simultaneous transfection. Lipoplexes of LF2000/mRNA (with a final mRNA amount of 0.2 µg) were transferred to the cells cultured in Opti‐MEM with a final volume of 500 µL. The lipoplexes were incubated with cells for 4 h or 24 h for uptake experiments, while transfection efficiency was determined after 24 h total incubation time at 37°C, 5% CO_2_. At the end of incubation (e.g., 4 h or 24 h), cells were rinsed with DPBS[‐] twice, before imaging by a confocal laser scanning microscope (C1si, Nikon Benelux, Belgium) equipped with a 10 × objective lens (Plan Apo, NA 0.45, Nikon Benelux, Belgium). To quantify cellular uptake or protein expression, cells were trypsinized and the cell pellet was harvested and suspended in flow buffer for measurements by flow cytometry (Cytoflex, Beckman Counter, Suarlée, Belgium).

Subsequent transfection. Single‐lipoplexes containing 0.1 µg eGFP mRNA or mCherry mRNA were subsequently administered to the cells with a time interval of 24 h. After 4 incubation, the single‐lipoplexes were replaced by fresh culture medium. After the following 20 h, mCherry mRNA single‐lipoplexes or eGFP mRNA single‐lipoplexes were applied to the cells respectively for the second transfection, eventually corresponding to an overall dose of 0.2 µg mRNA per well. Cells were collected for flow cytometry measurements after imaging.

Successive transfection. Mingle‐lipoplexes and single‐lipoplexes at an R/G ratio of 50/50% mCherry/eGFP were applied to the cells by one, two or four successive transfections, with a total amount of 0.2 µg mRNA (i.e., 1 × 0.2 µg, 2 × 0.1 µg or 4 × 0.05 µg mRNA). After 4 h incubation, cells were washed and incubated with fresh culture medium for 20 h before performing another round of transfection or proceeding to confocal imaging and flow cytometry at the end of the transfection series.

### Ex Vivo Transfection

The retina organoids were grown according to the established protocols.^[^ ^44^, ^45^ ^]^ In brief, wild type mouse embryonic stem cell‐Line ES‐E14TG2a, purchased from Sigma Aldrich were cultured in Nunc Delta 25 cm^2^ flasks which were coated with a 0.1% w/v solution of gelatin from porcine skin (FlukaTM, BioChemika) in standard conditions (37°C, 5% CO_2_ and 95% humidity).^[^ ^46^ ^]^ The maintenance medium to passage the stem cells contained DMEM with 15% (vol%, same as below) heat inactivated FBS, 1% GlutaMAX, 1% non‐essential amino acids and 0.1% *β*‐mercaptoethanol (0.1 m in PBS). Mouse Leukemia Inhibitory Factor (mLIF, 0.01%, AMS Biotechnology) and MEK inhibitor (1 × 10^−6^ m) were added fresh. To start the generation of the organoids, the cells were washed once with PBS before adding TrypLE Express (Gibco) for 1 min. The cell suspension was transferred to a 15 mL falcon tube containing 4 mL maintenance medium. For each organoid, 3000 cells were seeded in a Nunclon Sphera 96‐well U‐Bottom plate (Thermo Scientific, Karlsruhe, Germany) in 100 µL retinal differentiation medium containing GMEM with 1% non‐essential amino acids, 1% pyruvate, 1.5% Knockout Serum Replacement and 0.1% *β*‐mercaptoethanol. One day after spheroid formation (i.e., day 1), Matrigel (Corning) was added to each well in a final concentration of 2%. The differentiation was continued on day 7 by transferring the organoids to a 24‐well Suspension Culture Plate with 1 mL retinal maturation medium 1 (DMEM/F12, 1% N2 supplement and 1% penicillin/streptomycin) per well. From day 7, the organoids were incubated at 37°C, 5% CO_2_ and 40% O_2_. Following the approach,^[^ ^44^ ^]^ the organoids were cut into three pieces using forceps and a Nikon Stereoscope SMZ25 (Nikon Instruments) with fluorescence at cultivation day 10. Then, they were further differentiated using retinal maturation medium 2 containing DMEM/F12 with 1% N2 supplement, 1% penicillin/streptomycin, 10% FBS, 0.25% taurine and 0.5% all trans retinoic acid. To prevent the cut organoids from attaching, they were transferred to a new plate the next day. From day 14 on until the end of cultivation, the organoids were cultured with retinal maturation medium 2 without retinoic acid and transferred twice per week to a new 24‐well plate while placed on a shaker inside the incubator. For the transfection, mRNA lipoplexes were incubated with the organoids at day 22 of cultivation, with 50 µL lipoplexes containing a total of 1.0 µg mRNA per well (e.g., 1.0 µg of mCherry mRNA, 0.75 µg/0.25 µg of mCherry/eGFP mRNA, 0.5 µg/0.5 µg of mCherry/eGFP mRNA, 0.25 µg/0.75 µg of mCherry/eGFP mRNA, and 1.0 µg of eGFP mRNA) delivered by mingle‐ or single‐lipoplexes) for 24 h at 37°C, 5% CO_2_ and 95% humidity. At the end of incubation, the organoids were rinsed by PBS and then fixed by 2% PFA for confocal imaging on TCS SP5 using a 25 × 0.95 water immersion objective (Leica, Germany).

### In Vivo Transfection

Zebrafish (Danio rerio, strain AB/TL) were maintained and handled according to the guidelines from the Zebrafish Model Organism Database (http://zfin.org) and in compliance with the directives of the local animal welfare committee of Leiden University. Fertilization was performed by natural spawning at the beginning of the light period, and eggs were raised at 28.5°C in egg water (60 µg mL^−1^ Instant Ocean sea salts). Mingle‐ or single‐lipoplex formulations (0.2 ng mRNA in 2 nL for each embryo) were injected into the hindbrain ventricle of 48 h post fertilization (hpf) zebrafish embryos. Embryos were anesthetized in 0.01% tricaine and embedded in 0.4% agarose containing tricaine before injection. Then, embryos were removed from the agarose. At indicated time‐points after injection, embryos were embedded again and imaged using confocal microscopy. Confocal z‐stacks were captured on a Leica TCS SPE confocal microscope, using a 10 × air objective (HCX PL FLUOTAR) or a 40 × water‐immersion objective (HCX APO L). Laser intensity, gain, and offset settings were identical between stacks and sessions. Images were processed by using the Fiji distribution of Image J.

### Statistical Analysis

Results are shown as mean ± standard deviation. Experiments were performed at least in triplicate on independent days. Significance between the means of two groups was tested using 2‐way ANOVA with the software GraphPad Prism 7. Asterisks indicate statistical significance: * *p* < 0.05; ** *p* < 0.01; *** *p* < 0.001.

## Conflict of Interest

The authors declare no conflict of interest.

## Author Contributions

H.Z, K.R.. K.B. conceived and designed the project. H.Z., J.B., F.H.H, J.D., and A.M. performed the experiments and data analysis. J.B. assisted with microinjecting mRNA complexes into zebrafish embryos. F.H.H and F.S. assisted with retinal organoids preparation and imaging. K.B. and M.R. derived the statistical model and performed data analysis. All authors contributed to the writing of the manuscript.

## Supporting information

Supporting Information

## Data Availability

The data that support the findings of this study are available from the corresponding author upon reasonable request.
