# Supplementary material for: Together is Better: mRNA Co‐Encapsulation in Lipoplexes is Required to Obtain Ratiometric Co‐Delivery and Protein Expression on the Single Cell Level
Source: Adv Sci (Weinh). 2021 Dec 16;9(4):2102072. doi: 10.1002/advs.202102072 (PMC8811815; doi:10.1002/advs.202102072)
Supplement: Supplementary file 1 — Supporting Information [file ADVS-9-2102072-s001.pdf]

ADVANCED SCIENCE
Open Access

Supporting Information

for *Adv. Sci.,* DOI: 10.1002/advs.202102072

Together is Better: mRNA Co-encapsulation in Lipoplexes is Required to Obtain Ratiometric Co-delivery and Protein Expression on the Single Cell Level

Heyang Zhang, Jeroen Bussmann, Florian H Huhnke, Joke Devoldere, An-Katrien Minnaert, Wim Jiskoot, Friedhelm Serwane, Joachim Spatz, Magnus Röding, Stefaan C. De Smedt, Kevin Braeckmans,* Katrien Remaut**

Together is better: mRNA co-encapsulation in lipoplexes is required to obtain ratiometric co-delivery and protein expression on the single cell level

Heyang Zhang[1], Jeroen Bussmann[2], Florian H Huhnke[3], Joke Devoldere[1], An-Katrien Minnaert[1], Wim Jiskoot[2†], Friedhelm Serwane[3, 4, 5, 6, 7], Joachim Spatz[3, 4], Magnus Röding[8, 9], Stefaan C. De Smedt[1, 10]*, Kevin Braeckmans[1,11]*, Katrien Remaut[1, 10]*

[1]Laboratory of General Biochemistry and Physical Pharmacy, Faculty of Pharmaceutical Sciences, Ghent University, 9000 Ghent, Belgium

[2]Division of BioTherapeutics, Leiden Academic Center for Drug Research, Leiden University, 2333 CC Leiden, The Netherlands

[3]Max Planck Institute for Medical Research, Department of Cellular Biophysics, 70569 Stuttgart, Germany

[4]University of Heidelberg, 69120 Heidelberg, Germany

[5]Center for NanoScience, Ludwig-Maximilian-University Munich, D-80333 Munich, Germany

[6]Faculty of Physics, Ludwig-Maximilian-University, D-80539 Munich, Germany

[7]Munich Cluster for Systems Neurology (SyNergy), D-81377 Munich, Germany

[8]RISE Research Institutes of Sweden, Bioeconomy and Health, Agriculture and Food, 41276 Göteborg, Sweden

[9]Department of Mathematical Sciences, Chalmers University of Technology and University of Gothenburg, 41296 Göteborg, Sweden

[10]Cancer Research Institute Ghent (CRIG)

[11]Center for Advanced Light Microscopy, Ghent University, 9000 Ghent, Belgium

† In memoriam of Prof. Wim Jiskoot, who sadly passed away during the revision process of this manuscript.

E-mail: Katrien.Remaut@UGent.be; Kevin.Braeckmans@UGent.be; Stefaan.Desmedt@UGent.be

Tel: +32 9 264 80 76, Fax: +32 9 264 81 89

Corresponding address: Ottergemsesteenweg 460, 9000 Ghent, Belgium

Supporting Information

Part 1

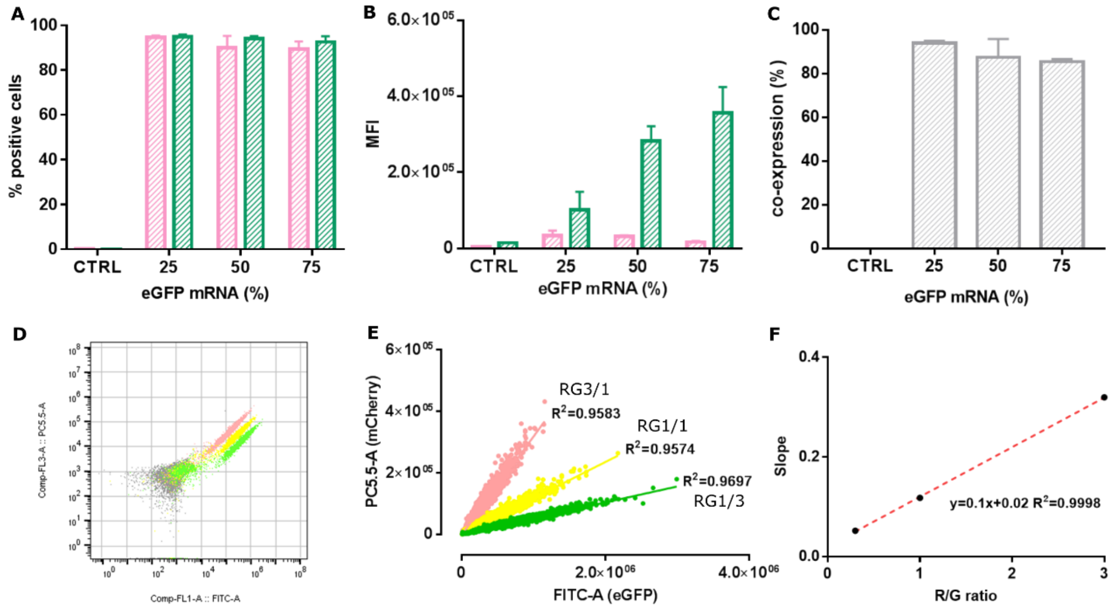

Supplementary Figure 1. The mixture of naked mRNA encoding mCherry (red bars) and eGFP (green bars) at a ratio of 75/25, 50/50 and 25/75%, was delivered to HeLa cells through nucleofection. After 24 hours' incubation, the HeLa cells were examined by flow cytometry for protein expression. (A) Percentage of positive cells, (B) mean fluorescence intensity (MFI), (C) fraction of positive cells expressing both mCherry and eGFP. X-axis represents the percentage of eGFP mRNA in the mixtures. (D) representative flow cytometry dot plots with applied red and green fluorescence threshold, (E) correlation between red and green fluorescence as derived from flow cytometry dot plots and (F) the slope of dot plots as a function of initial R/G mRNA ratio. All the data was averaged from three independent experiments, with three replicates per repeat (n=9). The total mRNA amount was kept constant at 2.0 µg/well.

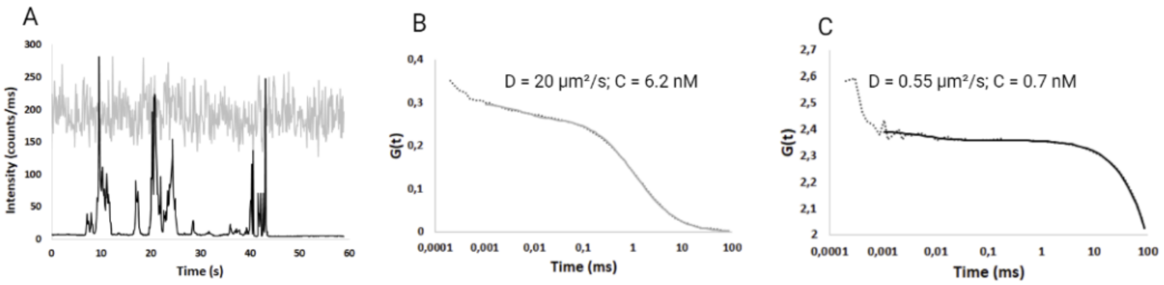

Supplementary Figure 2. Principle of single-color FCS auto-correlation analysis of number of mRNA per lipoplex. A. Fluorescence fluctuations of mRNA before (grey) and after (black) encapsulation in lipoplexes. Fluorescence peaks demonstrate encapsulation of multiple mRNAs per complex. B. Auto-correlation curve (dotted line) and fitting (solid line) of free mRNA before complexation . C. Auto-correlation (dotted line) and fitting (solid line) of mRNA containing lipoplexes. In this example, 6.2 nM red fluorescent mRNA was distributed over 0.7 nM lipoplexes, leading to 8.3 number of mRNA molecules per lipoplex, taking into account 93.4% encapsulation efficiency. It should be noted this is an average number, as the individual mRNA molecules per lipoplex will also depend on the lipoplex size and can be expected to be heterogeneous.

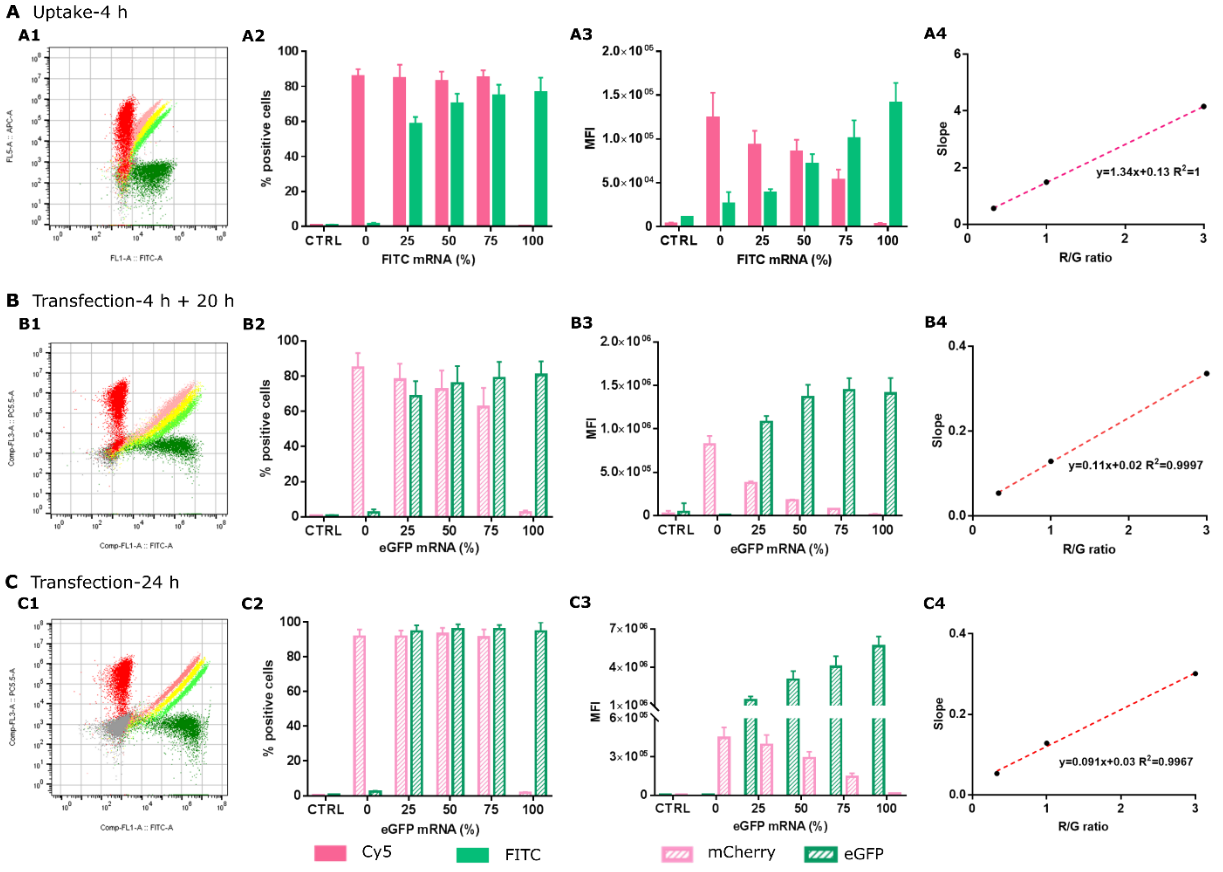

Supplementary Figure 3. (A) The mixture of Cy5 mRNA (red, R*) and FITC mRNA (green, G*) at an R*/G* ratio of 100/0, 75/25, 50/50, 25/75 and 0/100% was formulated into mingle-lipoplexes, before being applied to cells. The mingle-lipoplexes were incubated with HeLa cells for 4 h to examine the cellular uptake. (B, C) Mingle-lipoplexes that contain mCherry mRNA and eGFP mRNA at an R/G ratio of 100/0, 75/25, 50/50, 25/75 and 0/100% were incubated with HeLa cells. (B) After 4 hours' incubation, cells were rinsed and left to grow for another 20 h in fresh culture medium before flow cytometry measurements. (C) The mingle-lipoplexes were incubated with HeLa cells for 24 h to examine the transfection. At the end of incubation, cells were harvested for flow cytometry measurements. (A1, B1, C1) Representative flow cytometry dot plots with applied red and green fluorescence threshold, (A2, B2, C2) percentage of positive cells, (A3, B3, C3) MFI and (A4, B4, C4) the slope of dot plots as a function of initial R/G mRNA ratio. All the data was averaged from three independent experiments, with three replicates per repeat (n=9). The total mRNA amount was kept constant at 0.2 µg/well.

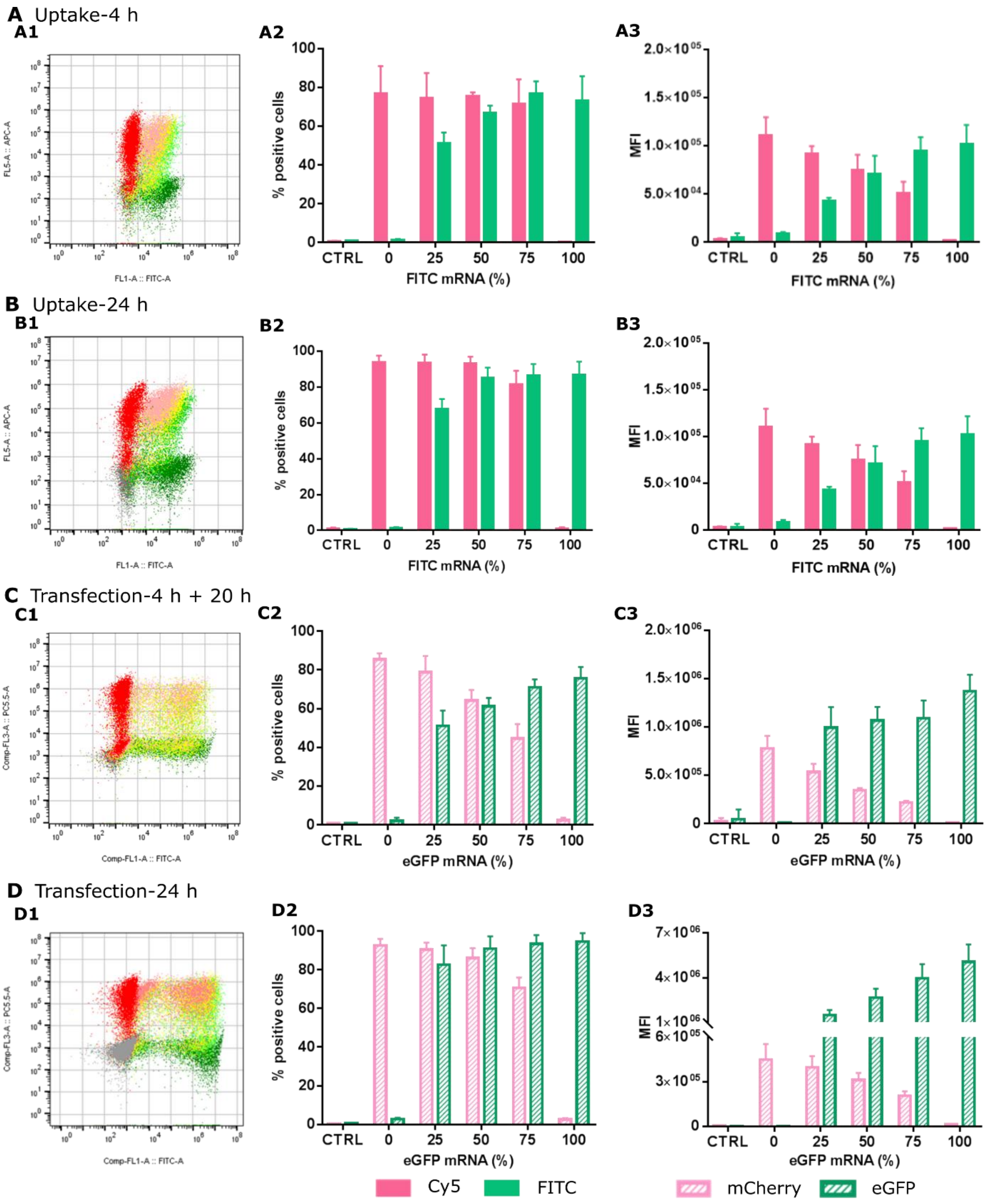

Supplementary Figure 4. (A, B) The mixture of Cy5 mRNA (red, R*) and FITC mRNA (green, G*) at an R*/G* ratio of 100/0, 75/25, 50/50, 25/75 and 0/100% was formulated into single-lipoplexes, before being applied to cells. The single-lipoplexes were incubated with HeLa cells for (A) 4 h and (B) 24 h to examine the cellular uptake. (C) Single-lipoplexes that contain mCherry mRNA and eGFP mRNA at an R/G ratio of 100/0, 75/25, 50/50, 25/75 and 0/100% were incubated with HeLa cells. (C) After 4 hours' incubation, cells were rinsed and

left to grow for another 20 h in fresh culture medium before flow cytometry measurements. (D) The single-lipoplexes were incubated with HeLa cells for 24 h to examine the transfection. At the end of incubation, cells were harvested for flow cytometry measurements. (A1, B1, C1, D1) Representative flow cytometry dot plots with applied red and green fluorescence threshold, (A2, B2, C2, D2) percentage of positive cells and (A3, B3, C3, D3) MFI. All the data was averaged from three independent experiments, with three replicates per repeat (n=9). The total mRNA amount was kept constant at 0.2 µg/well.

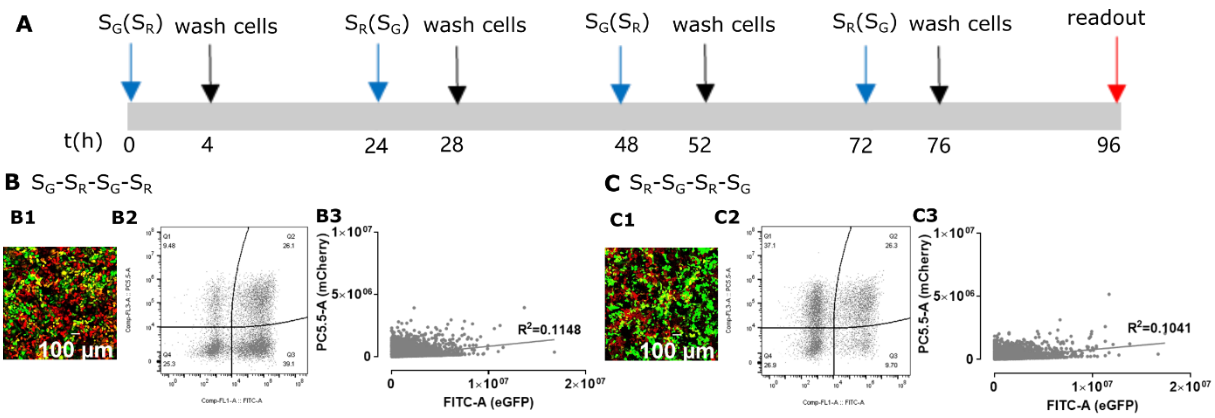

Supplementary Figure 5. (A) Subsequent transfection of eGFP mRNA single-lipoplexes and mCherry mRNA single-lipoplexes ((B) red follows green and (C) green follows red). After 4 hours' incubation, cells were rinsed and replaced with fresh culture medium and left to grow for another 20 h before next round of transfection. After 4 times transfection, cells were rinsed before confocal imaging and flow cytometry measurements. (B1, C1) Representative confocal images, (B2, C2) representative flow cytometry dot plots with applied red and green fluorescence threshold, and (B3, C3) correlation between red and green fluorescence as derived from (B2, C2) flow cytometry dot plots respectively. Scale bar: 100 µm. All the experiments were performed in triplicate. The mRNA dose was 0.05 µg/well per transfection round, leading to a total mRNA amount of 0.2 µg/well after the fourth transfection.

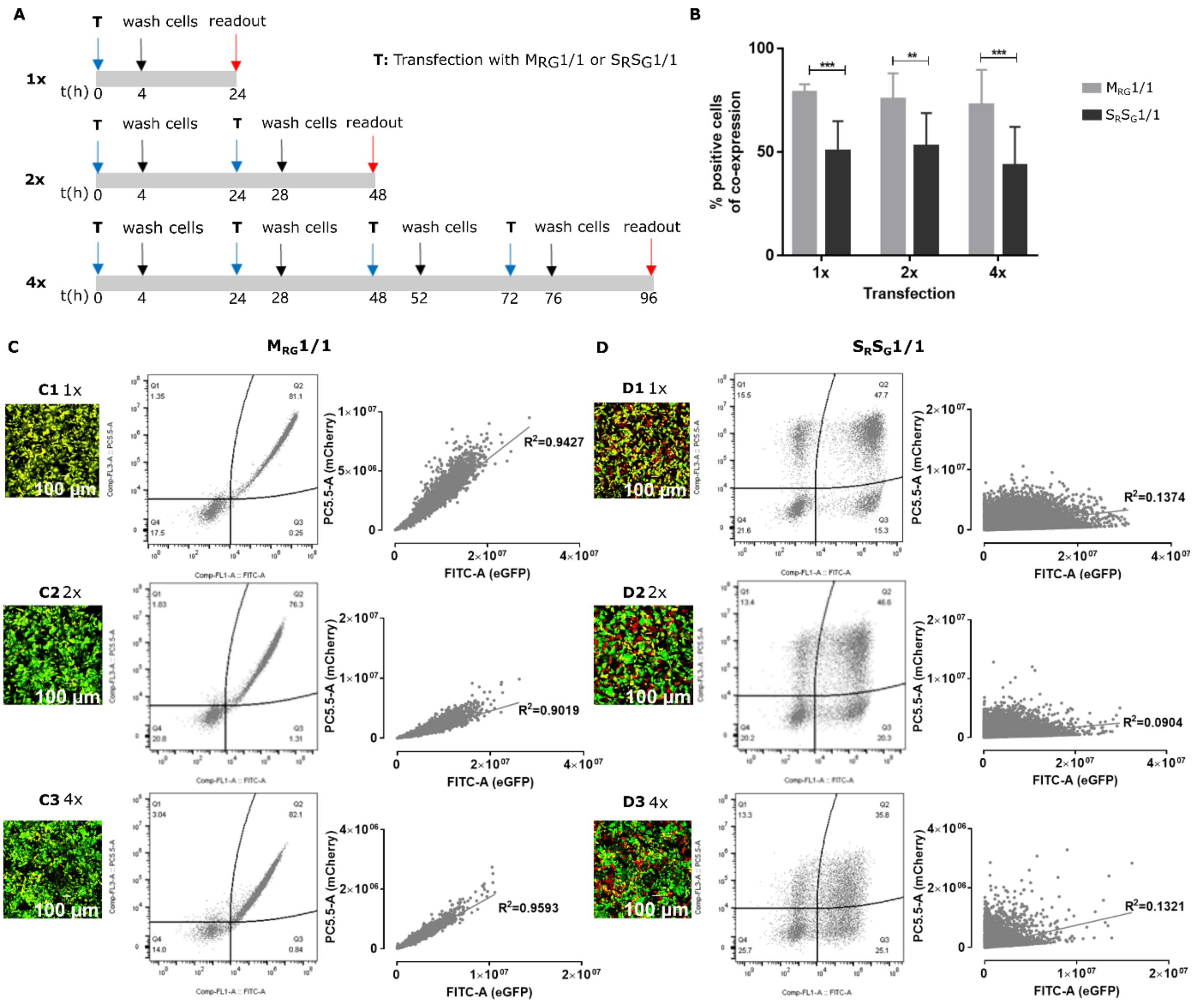

Supplementary Figure 6. Successive transfection of mRNA lipoplexes on HeLa cells. (A) Scheme of successive transfection of mRNA lipoplexes with a time interval of 24 h. Briefly, cells were rinsed twice and replaced by fresh culture medium after incubating with lipoplexes for 4 h, and left to grow for another 20 h before another round of transfection or before confocal imaging and flow cytometry measurements. (B) Quantification of positive cells (%) with co-expression of eGFP and mCherry protein after one, two and four successive transfections of mingle-lipoplexes (M_{RG}1/1) or single-lipoplexes (S_RS_G1/1) at a R/G ratio of 50/50%. (C, D) Representative confocal images and flow cytometry scatter plots of HeLa cells receiving one, two and four successive transfections with 50/50% (w/w) of mCherry/eGFP mRNA delivered by mingle-lipoplexes or single-lipoplexes respectively. All data was averaged from three independent experiments, with three replicates per repeat (n=9). Scale bar: 100 µm. The mRNA dose was 0.2, 0.1 or 0.05 µg/well per transfection round, leading to a total mRNA amount of 0.2 µg/well after respectively the first, second or fourth transfection.

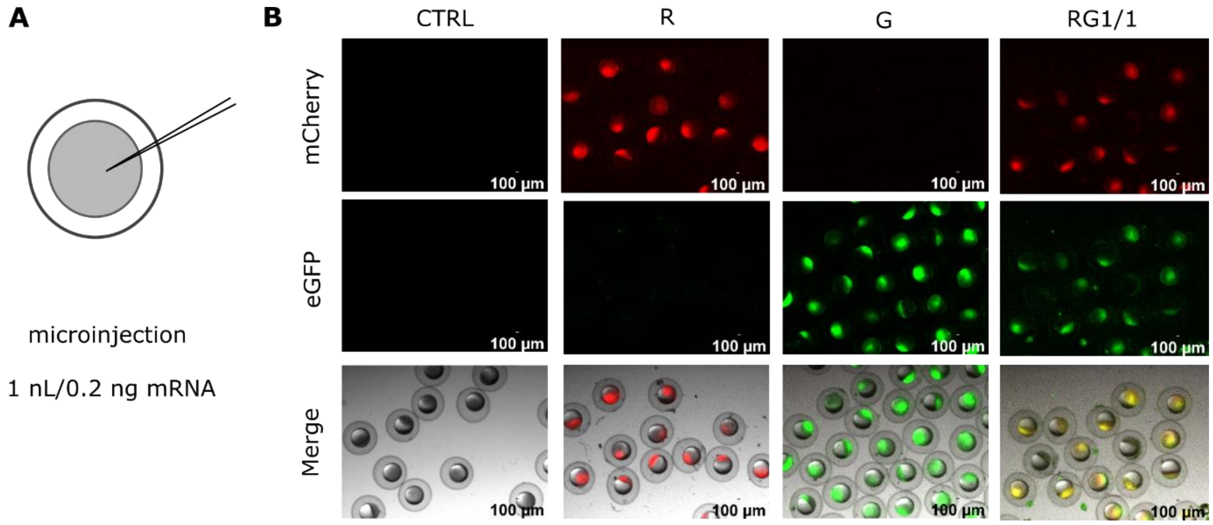

Supplementary Figure 7. (A) 1-cell stage zebrafish embryos transfected with naked mRNA encoding mCherry (red, R) and eGFP (green, G) through microinjection. (B) Representative images of zebrafish embryos at 1-cell stage after microinjection of 0.2 nL containing 0.2 ng mCherry mRNA, 0.1 ng/0.1 ng of mCherry mRNA/eGFP mRNA mixture, and 0.2 ng eGFP mRNA. The zebrafish embryos without treatment were used as control. After 24 h incubation, zebrafish embryos were embedded in agarose for imaging. Around 50 zebrafish embryos were injected for each sample. Scale bar: 100 µm.

Part 2 - Statistical description of double mRNA transfections by single- and mingle-lipoplexes

The aim is to give a theoretical derivation of the correlation of the expression of two types of mRNA molecules that are delivered to cells by single- and mingle-lipoplexes. As in the main text, we define single-lipoplexes as lipoplexes that contain only one type of cargo, while mingle-lipoplexes contain a mixture of different cargo molecules. We will start with the case of a single mRNA molecule and derive the distribution (often referred to as the probability mass function or pmf in the case of discrete-valued random variables) of the number of mRNA molecules that have reached the cytosol. We will consider the number of mRNA molecules per lipoplex, the association/uptake of lipoplexes into cells and the subsequent release of mRNA into the cytosol. Then we will expand this model to the case of two types of mRNA molecules delivered by single-lipoplexes and derive the joint distribution for uptake and cytosolic release. Next, we do the same for mingle-lipoplexes and consider expansion of the model towards protein expression, focusing in particular on deriving the correlation between the expression of both types of proteins. Finally, we consider how the model can be used to quantitatively interpret the experimental correlation values.

1. Distribution of the number of cytosolic cargo molecules per cell delivered by lipoplexes

We consider lipoplexes each containing m cargo molecules (e.g., mRNA), where m is constant. These lipoplexes associate with or internalize into cells (e.g., by endocytosis) after a certain incubation period. Let K be the number of lipoplexes in a cell (which depends on the applied concentration and incubation time), which is random with distribution $f_K(k)$. Of those, a certain (random) fraction will release their cargo successfully into the cell (e.g., by direct translocation over the cell membrane or by endosomal escape). Let X $(0 \leq X \leq K)$ be the number of lipoplexes that release their cargo into the cell, which is random with distribution $f_X(x)$. Consider a cell with $K = k$ lipoplexes and let the distribution of X under the condition that $K = k$, $X|K = k$, be $f_{X|K}(x; k)$. It follows that the distribution of X is a so-called compound distribution,

$$f_X(x) = \sum_{k=0}^{+\infty} f_K(k) f_{X|K}(x;k),$$

the mean (i.e., expectation) and variance of which can be calculated from the laws of total expectation and total variance,

$$\mathrm{E}[X] = \mathrm{E}\big[\mathrm{E}[X|K]\big]$$

$$\mathrm{Var}(X) = \mathrm{E}[\mathrm{Var}(X|K)] + \mathrm{Var}(\mathrm{E}[X|K])$$

The number of available cargo molecules per cell is $N = mX$, with distribution

$$f_N(n) = f_X\left(\frac{n}{m}\right).$$

It is worth noting that, whereas X can attain all non-negative integer values, N can only attain non-negative integer multiples of m. The mean and variance can be readily obtained from those for X,

$$\mathrm{E}[N] = m\mathrm{E}[X]$$

$$\mathrm{Var}(N) = m^2\mathrm{Var}(X)$$

If we consider successful release of the cargo molecules from a lipoplex to be a hit or miss process that occurs with probability p_e, then for a given $K = k$ the number of successfully released lipoplexes $X|K = k$ will follow a binomial distribution, $f_{X|K}(x;k) = \mathrm{Bino}(x;k,p_e)$ with $\mathrm{E}[X|K = k] = kp_e$ and $\mathrm{Var}(X|K = k) = kp_e(1 - p_e)$. The distribution of X is then

$$f_X(x) = \sum_{k=0}^{+\infty} f_K(k)\mathrm{Bino}(x;k,p_e)$$

with mean and variance

$$\mathrm{E}[X] = \mathrm{E}\big[\mathrm{E}[X|K]\big] = \mathrm{E}[Kp_e] = p_e\mathrm{E}[K]$$

$$\mathrm{Var}(X) = \mathrm{E}[\mathrm{Var}(X|K)] + \mathrm{Var}(\mathrm{E}[X|K]) = \mathrm{E}[Kp_e(1 - p_e)] + \mathrm{Var}(Kp_e)$$

$$= p_e(1 - p_e)\mathrm{E}[K] + p_e^2\mathrm{Var}(K)$$

For later use it is of interest to also determine the coefficient of dispersion $D(X)$ of $f_X(x)$,

$$D(X) = \frac{\mathrm{Var}(X)}{\mathrm{E}[X]} = \frac{p_e(1-p_e)\mathrm{E}[K] + p_e^2 \mathrm{Var}(K)}{p_e \mathrm{E}[K]} = 1 - p_e + p_e D(K) = 1 + p_e(D(K) - 1)$$

2. Joint distribution of the number of cytosolic cargo molecules per cell delivered by two types of single- lipoplexes

2.1 Joint distribution of the number of single-lipoplexes of type 1 and 2 associated per cell

Suppose we have two types of cargo molecules, each incorporated under exactly the same conditions into their own single-lipoplexes. In that case, the number of cargo molecules per lipoplex is the same for both types of single-lipoplexes, i.e., $m_1 = m_2 = m$. Consider the case where the two types of lipoplexes are added to cells in respective fractions α_1 and α_2 (with $\alpha_1 + \alpha_2 = 1$). Let K_1 and K_2 be the numbers of lipoplexes of the two types that are associated per cell. Note that the derivation performed above in section 1 is still true for the total number of lipoplexes $K = K_1 + K_2$. If we consider a cell with in total $K = k$ lipoplexes, then the probability of having k_1 lipoplexes of type 1 will be $f_{K_1|K}(k_1; k) = \text{Bino}(k_1; k, \alpha_1)$, or similarly $f_{K_2|K}(k_2; k) = \text{Bino}(k_2; k, \alpha_2)$ for type 2 lipoplexes. If we consider a cell with $K = k$ and $K_1 = k_1$, then the number of type 2 lipoplexes will be exactly $k_2 = k - k_1$, so that $f_{K_2|K,K_1}(k_2; k, k_1) = \mathbf{1}(k_2 = k - k_1)$, where $\mathbf{1}(k_2 = k - k_1)$ refers to the indicator function

$$\mathbf{1}(k_2 = k - k_1) = \begin{cases} 0 & \text{if } k_2 \neq k - k_1 \\ 1 & \text{if } k_2 = k - k_1 \end{cases}$$

The probability of having $K_1 = k_1$ lipoplexes of type 1 and $K_2 = k_2$ lipoplexes of type 2 given $K = k$ is then

$$f_{K_1,K_2|K}(k_1, k_2; k) = f_{K_2|K,K_1}(k_2; k, k_1) f_{K_1|K}(k_1; k) = \mathbf{1}(k_2 = k - k_1)\text{Bino}(k_1; k, \alpha_1).$$

The joint distribution $f_{K_1,K_2}(k_1, k_2)$ is then analogously to section 1 a compound distribution,

$$f_{K_1,K_2}(k_1, k_2) = \sum_{k=0}^{+\infty} f_K(k) f_{K_1,K_2|K}(k_1, k_2; k) = \sum_{k=0}^{+\infty} f_K(k)\mathbf{1}(k_2 = k - k_1)\text{Bino}(k_1; k, \alpha_1).$$

Note that completely complementary we could write that

$$f_{K_1,K_2}(k_1,k_2) = \sum_{k=0}^{+\infty} f_K(k)\mathbf{1}(k_1 = k - k_2)\text{Bino}(k_2;k,\alpha_2).$$

The marginal distributions are

$$f_{K_i}(k_i) = \sum_{k=0}^{+\infty} f_K(k)\text{Bino}(k_i;k,\alpha_i)$$

where the index $i = 1,2$ refers to the first or second type of cargo molecules (or corresponding single-lipoplexes). The marginal distributions have mean and variance

$$\mathrm{E}[K_i] = \mathrm{E}\big[\mathrm{E}[K_i|K]\big] = \mathrm{E}[\alpha_i K] = \alpha_i \mathrm{E}[K]$$

$$\begin{aligned}\mathrm{Var}(K_i) &= \mathrm{E}[\mathrm{Var}(K_i|K)] + \mathrm{Var}(\mathrm{E}[K_i|K]) = \mathrm{E}[K\alpha_i(1-\alpha_i)] + \mathrm{Var}(\alpha_i K)\\ &= \alpha_i(1-\alpha_i)\mathrm{E}[K] + \alpha_i^2\mathrm{Var}(K)\end{aligned}$$

The covariance and (Pearson) correlation of K_1 and K_2 can be calculated from the joint distribution $f_{K_1,K_2}(k_1,k_2)$ and the law of total covariance (Appendix 1):

$$\text{Cov}(K_1,K_2) = \alpha_1\alpha_2(\text{Var}(K) - E[K])$$

$$\text{Corr}(K_1,K_2) = \frac{\mathrm{D}(K) - 1}{\sqrt{\big(1+\rho_\alpha \mathrm{D}(K)\big)(1+\mathrm{D}(K)/\rho_\alpha)}}$$

where $\rho_\alpha = \frac{\alpha_1}{\alpha_2}$ and $D(K) = \frac{\text{Var}(K)}{\text{E}(K)}$ is the coefficient of dispersion of K.

For $\alpha_1 \to 0$ or $\alpha_1 \to 1$ the correlation obviously tends to 0, as one would expect. Note that even though the correlation can be negative, in practice we expect a positive value. This is because cells are exposed to lipoplexes in the cell culture medium for a given amount of time. As lipoplexes will be undergoing random Brownian diffusion, arrival and uptake of lipoplexes in cells can be reasonably assumed to follow a Poisson distribution. This is under the assumption of using a relatively low lipoplex concentration so that arrival of the next lipoplex to a cell is not hindered by previously

arrived lipoplexes. If $f_K(k) = \mathrm{Poisson}(k;\lambda)$, with λ the mean number of lipoplexes per cell, then obviously $D(K) = 1$. In reality, however, λ will not be a constant value for every cell, but will depend on cell size, cycle, and other factors that may change the uptake rate of a given cell. Therefore, for a given mean number of lipoplexes per cell $\mathrm{E}[K] = \lambda$ we expect that in reality $\mathrm{Var}(K) \geq \lambda$, so that indeed $D(K) \geq 1$. Supplementary Fig. 8 shows $\mathrm{Corr}(K_1, K_2)$ as a function of $D(K)$ for different values of ρ_α. Note that for a given value of $D(K)$ the correlation is maximal for $\rho_\alpha = 0.5$ and decreases symmetrically for lower and higher values of ρ_α.

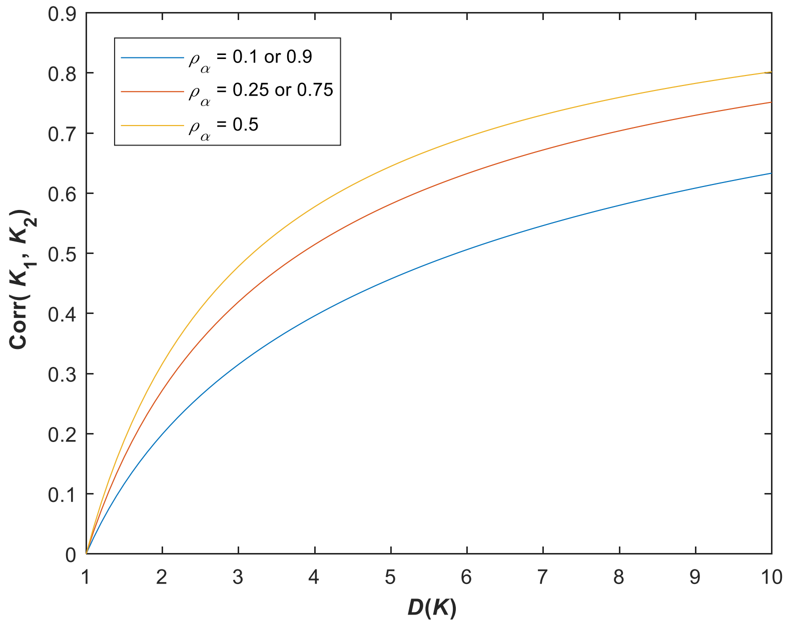

Supplementary Figure 8. The correlation $\mathrm{Corr}(K_1, K_2)$ between the number of lipoplexes of type 1 and 2 associated per cell is shown as a function of the dispersion coefficient $D(K)$ of the distribution of the total number of lipoplexes per cell for different ratios ρ_α.

2.2 Joint distribution of the number of cargo molecules of type 1 and 2 associated per cell

Experimentally we have not measured uptake of lipoplexes directly, but rather uptake of (labeled) cargo molecules. Given that we have m cargo molecules in each lipoplex, let $L_1 = mK_1$ and $L_2 = mK_2$ denote the number of cargo molecules of type 1 and type 2 in a cell. Then, the corresponding joint distribution is

$$f_{L_1,L_2}(l_1,l_2) = \sum_{k=0}^{+\infty} f_K(k)\mathbf{1}\left(\frac{l_2}{m} = k - k_1\right)\text{Bino}\left(\frac{l_1}{m};k,\alpha_1\right)$$

$$= \sum_{k=0}^{+\infty} f_K(k)\mathbf{1}(l_2 = mk - l_1)\text{Bino}\left(\frac{l_1}{m};k,\alpha_1\right)$$

with marginal distributions

$$f_{L_i}(l_i) = \sum_{k=0}^{+\infty} f_K(k)\text{Bino}\left(\frac{l_i}{m};k,\alpha_i\right)$$

having mean and variance

$$\mathrm{E}[L_i] = m\mathrm{E}[K_i] = m\alpha_i\mathrm{E}[K]$$

$$\mathrm{Var}(L_i) = m^2\mathrm{Var}(K_i) = m^2\alpha_i\big((1-\alpha_i)\mathrm{E}[K] + \alpha_i\mathrm{Var}(K)\big)$$

The covariance and correlation become

$$\text{Cov}(L_1,L_2) = \text{Cov}(mK_1,mK_2) = m^2\text{Cov}(K_1,K_2) = m^2\alpha_1\alpha_2(\mathrm{Var(K)} - \mathrm{E}[K])$$

$$\mathrm{Corr}(L_1,L_2) = \frac{\text{Cov}(L_1,L_2)}{\sqrt{\mathrm{Var}(L_1)\mathrm{Var}(L_2)}} = \frac{m^2\text{Cov}(K_1,K_2)}{\sqrt{m^2\mathrm{Var}(K_i)}} = \text{Corr}(K_1,K_2)$$

Thus we find that the correlation is the same for the number of associated lipoplexes and cargo molecules.

2.3 Joint distribution of the number of lipoplexes of type 1 and 2 per cell that successfully release their cargo into the cytosol

Now we can proceed with determining the joint distribution of the number of cargo molecules that are successfully released in the cell's cytosol. Let the total number of lipoplexes that release their cargo in a cell be X as before. If we consider a cell in which $X = x$ lipoplexes release their cargo, then the probability that x_1 of those is of type 1 will be $f_{X_1|X}(x_1|x) = \text{Bino}(x_1;x,\alpha_1)$. For $X = x$ and $X_1 = x_1$, the number of type 2 lipoplexes will then be exactly $x_2 = x - x_1$, so that $f_{X_2|X,X_1}(x_2|x,x_1) = \mathbf{1}(x_2 = x - x_1)$. We then have the joint distribution

$$f_{X_1,X_2}(x_1,x_2) = \sum_{x=0}^{+\infty} f_X(x) f_{X_2|X,X_1}(x_2;x,x_1) f_{X_1|X}(x_1;x) = \sum_{x=0}^{+\infty} f_X(x)\mathbf{1}(x_2 = x - x_1)\mathrm{Bino}(x_1;x,\alpha_1)$$

with marginal distributions

$$f_{X_i}(x_i) = \sum_{x=0}^{+\infty} f_X(x)\mathrm{Bino}(x_i;x,\alpha_i)$$

having mean and variance:

$$\mathrm{E}[X_i] = \alpha_i \mathrm{E}[X]$$

$$\mathrm{Var}(X_i) = \alpha_i(1-\alpha_i)\mathrm{E}[X] + \alpha_i^2 \mathrm{Var}(X)$$

The covariance and correlation are calculated completely analogous to Appendix 1, leading to:

$$\mathrm{Cov}(X_1,X_2) = \alpha_1\alpha_2(\mathrm{Var}(X) - \mathrm{E}[X])$$

$$\mathrm{Corr}(X_1,X_2) = \frac{\mathrm{Cov}(X_1,X_2)}{\sqrt{\mathrm{Var}(X_1)\mathrm{Var}(X_2)}} = \frac{D(X)-1}{\sqrt{(1+D(X)\rho_\alpha)(1+D(X)/\rho_\alpha)}}$$

Note that this is exactly the same expression as derived above for $\mathrm{Corr}(K_1,K_2)$, the only difference being that $D(K)$ is replaced by $D(X) = \frac{\mathrm{Var}(X)}{\mathrm{E}[X]}$, the coefficient of dispersion of X. Note that also here we expect a positive correlation, since realistically $D(X) > 1$. This is because $D(X) = 1 + p_e(D(K)-1)$, as we derived before, so that $D(X) \geq 1$ if $D(K) \geq 1$, which is indeed the case as we have already established before. It also follows from the same relation that $D(X) \leq D(K)$, so that $\mathrm{Corr}(X_1,X_2) \leq \mathrm{Corr}(K_1,K_2)$ due to the fact that the correlation is a monotonically increasing function of the coefficient of dispersion. Note that $\mathrm{Corr}(X_1,X_2) \to \mathrm{Corr}(K_1,K_2)$ for $p_e \to 1$. An example for $p_e = 0.15$ is shown in Supplementary Fig. 9 where $\mathrm{Corr}(X_1,X_2)$ is plotted as a function of $D(K)$. Direct comparison with Supplementary Fig. 9 indeed shows that the correlation of cytosolically released cargo molecules is less than for the cell associated cargo molecules for the same values of ρ_α.

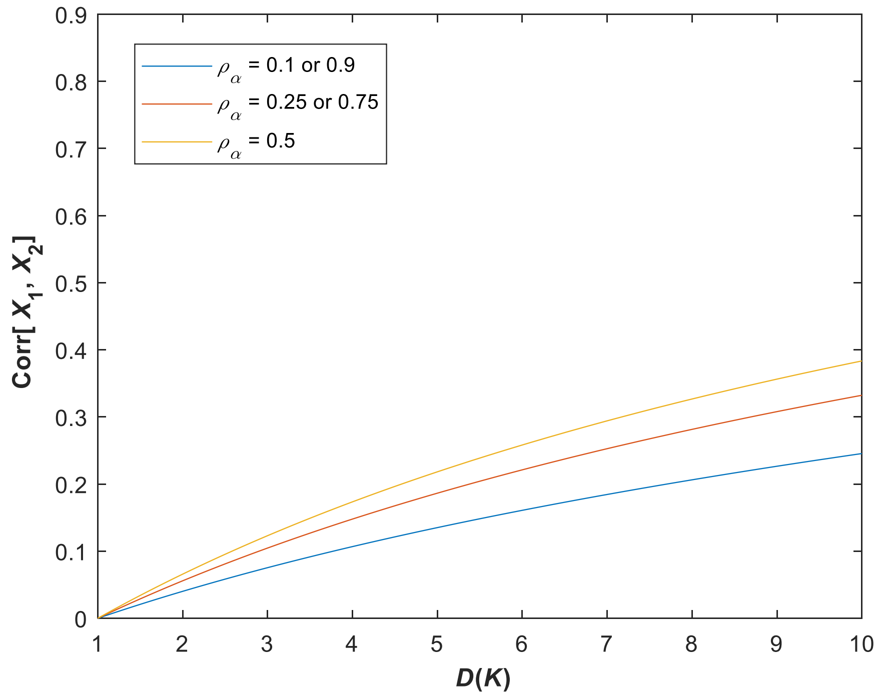

Supplementary Figure 9. The correlation $\mathrm{Corr}(X_1, X_2)$ between the number of cytosolic cargo molecules of type 1 and 2 associated per cell is shown as a function of the dispersion $D(K)$ for different ratios ρ_α. For the cytosolic release probability a value of $p_e = 0.15$ was chosen.

2.4 Joint distribution of the number of cargo molecules of type 1 and 2 per cell that successfully reach the cytosol

Since the number of cytosolically released cargo molecules of type 1 and 2 is respectively $N_1 = mX_1$ and $N_2 = mX_2$, we then find that

$$f_{N_1,N_2}(n_1, n_2) = \sum_{x=0}^{+\infty} f_X(x)\mathbf{1}\left(\frac{n_2}{m} = x - x_1\right) \mathrm{Bino}\left(\frac{n_1}{m}; x, \alpha_1\right)$$

$$= \sum_{x=0}^{+\infty} f_X(x)\mathbf{1}(n_2 = mx - n_1)\mathrm{Bino}\left(\frac{n_1}{m}; x, \alpha_1\right)$$

with marginal distributions:

$$f_{N_i}(n_i) = \sum_{x=0}^{+\infty} f_X(x)\mathrm{Bino}\left(\frac{n_i}{m}; x, \alpha_i\right)$$

having mean and variance

$$\mathrm{E}[N_i] = m\mathrm{E}[X_i] = m\alpha_i\mathrm{E}[X]$$

$$\mathrm{Var}(N_i) = m^2\mathrm{Var}(X_i) = m^2\alpha_i\big((1-\alpha_i)\mathrm{E}[X] + \alpha_i\mathrm{Var}(X)\big)$$

The covariance and correlation are immediately obtained as

$$\mathrm{Cov}(N_1, N_2) = \mathrm{Cov}(mX_1, mX_2) = m^2\mathrm{Cov}(X_1, X_2)$$

$$\mathrm{Corr}(N_1, N_2) = \frac{\mathrm{Cov}(N_1, N_2)}{\sqrt{\mathrm{Var}(N_1)\mathrm{Var}(N_2)}} = \mathrm{Corr}(X_1, X_2)$$

showing that the correlation remains unaltered whether one considers the number of released lipoplexes or cargo molecules.

Since $D(X) \leq D(K)$ it follows that that $\mathrm{Corr}(N_1, N_2) \leq \mathrm{Corr}(L_1, L_2)$, i.e., the correlation between the cargo molecules released in the cytosol must be less than the correlation of the cell-associated cargo molecules. Mathematically this is due to the fact that the derived correlation functions are essentially the same monotonic increasing function of the coefficient of dispersion D. Importantly and interestingly this corresponds to our experimental observation that the correlation of protein expression (which is proportional to the number of released cargo molecules) was less than the correlation of cargo uptake.

3. Joint distribution of the number of cytosolic cargo molecules per cell delivered by mingle-lipoplexes

3.1 Joint distribution of the number of cell-associated cargo molecules of type 1 and 2 delivered by mingle- lipoplexes

Consider mingle-lipoplexes prepared from a mixture of type 1 and type 2 cargo molecules with relative fractions α_1 and α_2. As before we take a constant number m of cargo molecules per lipoplex. Then the number M_1 of type 1 molecules per lipoplex follows a binomial distribution according to $f_{M_1}(m_1) = \mathrm{Bino}(m_1; m, \alpha_1)$, while the corresponding number $M_2 = m_2$ of type 2 molecules for

$M_1 = m_1$ will be exactly $m_2 = m - m_1$ so that $f_{M_2|M_1}(m_2; m_1) = \mathbf{1}(m_2 = m - m_1)$. The joint distribution of M_1 and M_2 then is

$$f_{M_1,M_2}(m_1, m_2) = f_{M_2|M_1}(m_2|m_1) f_{M_1}(m_1) = \mathbf{1}(m_2 = m - m_1)\text{Bino}(m_1; m, \alpha_1)$$

Assume now that K lipoplexes have associated with a given cell, corresponding to a total of $L = mK$ cargo molecules, of which L_1 and L_2 are of type 1 and 2, respectively. For $K = k$, if we denote the number of type 1 molecules in the jth lipoplex as M_{1j} $(j = 1, \dots, k)$, then for the total cell-associated cargo molecules of type 1 and type 2 we have

$$L_1 = \sum_{j=1}^{k} M_{1j}$$

$$L_2 = mk - L_1$$

Since L_1 is the sum of k independent and identically distributed binomial variables, we obtain

$$f_{L_1|K}(l_1; k) = \text{Bino}(l_1; km, \alpha_1)$$

$$f_{L_2|K,L_1}(l_2; k, l_1) = \mathbf{1}(l_2 = km - l_1)$$

leading to the joint distribution

$$f_{L_1,L_2|K}(l_1, l_2; k) = f_{L_2|K,L_1}(l_2; k, l_1) f_{L_1|K}(l_1; k) = \mathbf{1}(l_2 = km - l_1)\text{Bino}(l_1; km, \alpha_1)$$

with corresponding marginal distributions

$$f_{L_i|K}(l_i; k) = \text{Bino}(l_i; km, \alpha_i)$$

having mean and variance

$$\mathrm{E}[L_i|K = k] = k\mathrm{E}[M_i] = k\alpha_i m$$

$$\mathrm{Var}(L_i|K = k) = k\mathrm{Var}(M_i) = k\alpha_i(1 - \alpha_i)m$$

The joint distribution of internalized cargo molecules of type 1 and 2 is then

$$f_{L_1,L_2}(l_1,l_2) = \sum_{k=0}^{+\infty} f_K(k) f_{L_1,L_2|K}(l_1,l_2;k) = \sum_{k=0}^{+\infty} f_K(k)\mathbf{1}(l_2 = km - l_1)\text{Bino}(l_1; km, \alpha_1)$$

with corresponding marginal distributions

$$f_{L_i}(l_i) = \sum_{k=0}^{+\infty} f_K(k)\text{Bino}(l_1; km, \alpha_1)$$

having mean and variance:

$$\mathrm{E}[L_i] = \mathrm{E}\big[\mathrm{E}[L_i|K]\big] = \mathrm{E}[K\alpha_i m] = \alpha_i m\mathrm{E}[K]$$

$$\begin{aligned}\mathrm{Var}(L_i) &= E[\mathrm{Var}(L_i|K)] + \mathrm{Var}(\mathrm{E}[L_i|K]) = E[K\alpha_i(1-\alpha_i)m] + Var(K\alpha_i m) \\ &= \alpha_i(1-\alpha_i)m\mathrm{E}[K] + (\alpha_i m)^2\mathrm{Var}(K) = \alpha_i m\big((1-\alpha_i)\mathrm{E}[K] + \alpha_i m\mathrm{Var}(K)\big)\end{aligned}$$

The covariance and correlation are (Appendix 2):

$$\mathrm{Cov}(L_1, L_2) = \alpha_1\alpha_2 m(m\mathrm{Var}(K) - \mathrm{E}[K])$$

$$\mathrm{Corr}(L_1, L_2) = \frac{mD(K) - 1}{\sqrt{\big(1 + m\rho_\alpha D(K)\big)(1 + m\,D(K)/\rho_\alpha)}}$$

where as before we defined the coefficient of dispersion $D(K) = \frac{\mathrm{Var}(K)}{\mathrm{E}[K]}$ and the ratio $\rho_\alpha = \frac{\alpha_1}{\alpha_2}$. Contrary to the single-lipoplex case, we now find that the correlation of cargo internalization depends on the number m of cargo molecules per particle. As a matter of fact, the expression for the correlation follows from the expression of single-lipoplexes with the substitution $D(K) \to mD(K)$, with the integer $m \geq 1$. Since the correlation for single-lipoplexes is monotonically increasing as a function of $D(K)$, it is clear that the correlation for mingle-lipoplexes will always be larger for all values $m > 1$ (or equal if $m = 1$). For the same reason we see that the correlation of cell-associated cargo delivered by mingle-lipoplexes increases with m, asymptotically approaching 1 as $m \to +\infty$.

Note that, even though the correlation for mingle-lipoplexes can theoretically be negative, in practice we only expect positive correlation values. Indeed, we have already argued that $D(K) \geq 1$, so that $mD(K) \geq 1$ as well.

3.2 Joint distribution of the number of cytosolically released cargo molecules of type 1 and 2 delivered by mingle-lipoplexes

Finally we will determine the joint distribution for cytosolically released cargo molecules of type 1 and type 2, the derivation of which is entirely analogous to the cell-associated cargo case which we just considered. Assume that $X = x$ lipoplexes can deliver their cargo molecules in the cytosol of a given cell, so that the total number of released cargo molecules is $N = n = mx$. If we denote the number of type 1 molecules in the jth released lipoplex as M_{1j}, then for the released cargo molecules of type 1 and type 2 we have:

$$N_1 = \sum_{j=1}^{x} M_{1j}$$

$$N_2 = mx - N_1$$

Thus, for $X = x$ the probability of having in total $N_1 = n_1$ molecules of type 1 and $N_2 = n_2$ molecules of type 2 in a cell's cytosol is:

$$f_{N_1,N_2|X}(n_1, n_2; x) = f_{N_2|X,N_1}(n_2; x, n_1) f_{N_1|X}(n_1; x, n) = \mathbf{1}(n_2 = mx - n_1)\text{Bino}(n_1; mx, \alpha_1)$$

with corresponding marginal distributions

$$f_{N_i|X}(n_i|x) = \text{Bino}(n_i; mx, \alpha_i)$$

having mean and variance:

$$\text{E}[N_i|X = x] = \alpha_i mx$$

$$\text{Var}(N_i|X = x) = \alpha_i mx(1 - \alpha_i)$$

If we let X have distribution $f_X(x)$ we finally obtain

$$f_{N_1,N_2}(n_1,n_2) = \sum_{x=0}^{+\infty} f_X(x) f_{N_1,N_2|X}(n_1,n_2;x) = \sum_{x=0}^{+\infty} f_X(x)\mathbf{1}(n_2 = mx - n_1)\text{Bino}(n_1;mx,\alpha_1)$$

for which, marginally,

$$f_{N_i}(n_i) = \sum_{x=0}^{+\infty} f_X(x)\text{Bino}(n_i;mx,\alpha_i)$$

with mean and variance

$$\mathrm{E}[N_i] = \mathrm{E}[\alpha_i mX] = \alpha_i m\mathrm{E}[X]$$

$$\text{Var}(N_i) = \mathrm{E}[\alpha_i(1-\alpha_i)mX] + \text{Var}(\alpha_i mX) = \alpha_i(1-\alpha_i)m\mathrm{E}[X] + (\alpha_i m)^2\text{Var}(X)$$

The covariance and correlation follow from an identical calculation as in Appendix 2:

$$\text{Cov}(N_1,N_2) = \alpha_1\alpha_2 m(m\text{Var}(X) - \mathrm{E}[X])$$

$$\text{Corr}(N_1,N_2) = \frac{mD(X)-1}{\sqrt{\big(1+m\rho_\alpha D(X)\big)(1+m\,D(X)/\rho_\alpha)}} = \text{Corr}(X_1,X_2)$$

For the same reason as explained for single-lipoplexes we have that $\text{Corr}(N_1,N_2) \le \text{Corr}(L_1,L_2)$, which corresponds to our experimental observations when comparing the correlation of uptake and expression data. And as noted in relation to the correlation of cargo uptake by mingle complexes, also $\text{Corr}(N_1,N_2)$ for mingle- lipoplexes will always be positive and larger than the correlation of released cargo by single-lipoplexes, which is precisely what we have observed experimentally as well.

Due to that

$$D(X) = 1 + p_e(D(K)-1)$$

also here we have that $\text{Corr}(N_1,N_2)$ can be expressed as a function of $D(K)$ and the probability of intracellular cargo release p_e. A simultaneous fit of $\text{Corr}(N_1,N_2)$ and $\text{Corr}(L_1,L_2)$ to their respective experimental data could, therefore, give an experimental estimate of p_e (and m). As a matter of fact, it would be possible to fit all uptake and expression data for both single and mingle-lipoplexes at

once since we expect the distribution of K, and hence $D(K)$, to be constant for all performed experiments. This is due to the fact that identical lipoplex concentrations and incubation times were used in all experiments and the fact that lipoplex uptake can reasonably be expected to be independent of the mRNA sequence used.

4. Conversion to fluorescence detection

4.1 Quantification of association of lipoplexes with cells

Experimentally we do not determine the number of cell-associated cargo molecules L_1 and L_2 directly, but rather quantify the fluorescence intensities I_1 and I_2 of labeled cargo molecules by flow cytometry. We assume that the fluorescence intensities are linear functions of the number of labeled cargo molecules, so that

$$I_1 = a_1 L_1 + b_1$$

$$I_2 = a_2 L_2 + b_2$$

Here, a_i $(i = 1, 2)$ is a proportionality constant that depends on the number of fluorophores per cargo molecule, fluorophore brightness and instrument settings, and b_i is a background term that account for autofluorescence and/or a detector offset. It should be noted that the $a_i$coefficient is time dependent and also depends on mRNA stability as well as protein stability. For simplicity we consider a_i and b_i to be constants. The joint distribution of I_1 and I_2 then readily follows from the corresponding expressions for the joint distribution of cell-associated cargo molecules:

$$f_{I_1,I_2}(i_1, i_2) = f_{L_1,L_2}\left(l_1 = \frac{i_1 - b_1}{a_1}, l_2 = \frac{i_2 - b_2}{a_2}\right)$$

Similarly, the corresponding marginal distributions are

$$f_{I_j}(i_j) = f_{L_j}\left(l_j = \frac{i_j - B_j}{A_j}\right)$$

with mean and variance

$$\mathrm{E}[I_j] = a_j \mathrm{E}[L_j] + b_j$$

$$\mathrm{Var}(I_j) = a_j^2 \mathrm{Var}(L_j)$$

For the covariance and correlation one readily obtains

$$\mathrm{Cov}(I_1, I_2) = \mathrm{Cov}(a_1 L_1 + b_1, a_2 L_2 + b_2) = \mathrm{Cov}(a_1 L_1, a_2 L_2) = a_1 a_2 \mathrm{Cov}(L_1, L_2)$$

$$\mathrm{Corr}(I_1, I_2) = \frac{a_1 a_2 \mathrm{Cov}(L_1, L_2)}{\sqrt{a_1^2 \mathrm{Var}(L_1) a_2^2 \mathrm{Var}(L_2)}} = \frac{\mathrm{Cov}(L_1, L_2)}{\sqrt{\mathrm{Var}(L_1)\mathrm{Var}(L_2)}} = \mathrm{Corr}(L_1, L_2)$$

Thus we find that the correlation of the fluorescence of labeled cargo molecules is identical to the correlation of the number of cell-associated cargo molecules so that the expressions for the correlation derived for single and mingle complexes remain valid.

4.2 Quantification of fluorescent protein expression

With regard to cargo release, so far we have considered the joint distribution and correlation of the *number* of cargo molecules of type 1 and type 2. In our experiments, however, we rather measure fluorescence protein expression (by flow cytometry). If we reasonably assume that protein expression and its fluorescence detection by flow cytometry is proportional to the number of cytosolic mRNA molecules, we can again write for the measured fluorescence intensity for each type of protein that

$$I_1 = a_1 N_1 + b_1$$

$$I_2 = a_2 N_2 + b_2$$

where a_i depends on the mRNA translation rate, fluorophore brightness and instrument settings, and b_i is still a background term that accounts for autofluorescence and/or detector offset. As before we consider a_i and b_i to be constants. Entirely analogous to the previous section we have that the joint distribution for protein expression by cytosolic cargo molecules is

$$f_{I_1, I_2}(i_1, i_2) = f_{N_1, N_2}\left(n_1 = \frac{i_1 - b_1}{a_1}, n_2 = \frac{i_2 - b_2}{a_2}\right)$$

The corresponding marginal distributions are

$$f_{I_j}(i_j) = f_{N_j}\left(n_j = \frac{i_j - b_j}{a_j}\right)$$

with mean, variance, covariance, and correlation

$$\mathrm{E}[I_j] = a_j \mathrm{E}[N_j] + b_j$$

$$\mathrm{Var}(I_j) = a_j^2 \mathrm{Var}(N_j)$$

$$\mathrm{Cov}(I_1, I_2) = a_1 a_2 \mathrm{Cov}(N_1, N_2)$$

$$\mathrm{Corr}(I_1, I_2) = \mathrm{Corr}(N_1, N_2)$$

Thus we find once more that the correlation of protein expression is identical to the correlation of the number of released cargo molecules so that the expressions for the correlation derived for single and mingle-lipoplexes remain valid.

5. Examples

The correlation functions $\mathrm{Corr}(I_1, I_2)$ are shown for $\alpha_1 = \alpha_2 = 0.5$, $m = 5$ and $p_e = 0.3$ in Supplementary Fig. 10. They confirm our experimental observations that for a given $\mathrm{D}(K)$ the correlation decreases when the cargo molecules are cytosolically released (compare full and dashed lines) or when the cargo molecules are delivered by single instead of mingle-lipoplexes (compare red with blue lines).

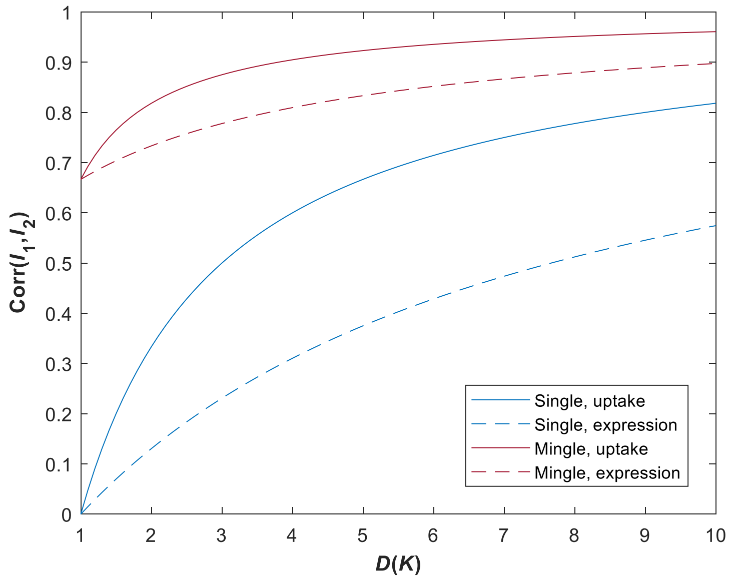

Supplementary Figure 10. The correlation functions for the four considered cases are shown as a function of $\mathrm{D}(K)$ for $\alpha_1 = \alpha_2 = 0.5$, $m = 5$ and $p_e = 0.3$. Solid lines correspond to cell uptake, while dashed lines are for cytosolically released cargo molecules (leading to protein expression). The blue and red curves correspond to single and mingle-lipoplexes, respectively.

In addition, we reckoned it would be instructive to visualize the above derived joint distributions for lipoplex uptake and protein expression by single- and mingle-complexes as well. This requires, however, a particular choice for the distribution $f_K(k)$ that describes lipoplex association to cells. As reported before[23], nanoparticle association to cells can be pragmatically described by an overdispersed Poisson distribution in the following fashion. Assume that $K|\Lambda = \lambda$ is Poisson distributed with mean $\mathrm{E}[K|\Lambda = \lambda] = \lambda$, and that Λ is gamma distributed with shape parameter κ and scale parameter θ. The resulting distribution of K is a negative binomial distribution which can be written as[1]:

$$f_K(k) = \mathrm{NegBino}(k; \kappa, \theta) = \frac{\Gamma(k+\kappa)}{k!\,\Gamma(\kappa)\theta^{\kappa}\left(1+\frac{1}{\theta}\right)^{\kappa+k}}$$

[1] Note that by defining $\theta = \frac{1-p}{p}$, this readily converts to the equivalent more common expression of the negative binomial distribution:

$$\mathrm{NegBino}(k; \kappa, p) = \frac{\Gamma(k+\kappa)}{k!\,\Gamma(\kappa)} p^{\kappa}(1-p)^{k}$$

The mean and variance are $\mathrm{E}[K] = \kappa\theta$ and $\mathrm{Var}(K) = \kappa\theta(1+\theta)$. The coefficient of dispersion is hence

$$D(K) = 1 + \theta$$

If one would increase the nanoparticle concentration or the incubation time, this would lead to a proportional increase of the average number of cell-associated lipoplexes, i.e., $\mathrm{E}[K]$. This is equivalent to an equal increase of $\mathrm{E}[\Lambda]$. This would in turn imply an increase of κ or θ, but since this is essentially a linear rescaling of Λ, primarily the scale parameter θ would increase. Hence, an increase of $\mathrm{E}[K]$ would lead to an increase of $D(K)$ as well. Due to the monotonicity of the correlation functions, they will increase too. This is in line with our experimental observation that the correlation increased for longer particle incubation times (see main text).

The distribution that describes the total number of cytosolic cargo molecules then becomes:

$$f_X(x) = \sum_{k=0}^{+\infty} f_K(k)\mathrm{Bino}(x; k, p_e) = \sum_{k=0}^{+\infty} \mathrm{NegBino}(k; \kappa, \theta)\mathrm{Bino}(x; k, p_e)$$

For clarity and convenience we here write the resulting joint distributions for all four cases:

1. Cell-associated cargo delivered by single-lipoplexes:

$$f_{I_1,I_2}(i_1, i_2) = \sum_{k=0}^{+\infty} \mathrm{NegBino}(k; \kappa, \theta)\mathbf{1}\left(\frac{i_2 - b_2}{a_2} = mk - \frac{i_1 - b_1}{a_1}\right)\text{Bino}\left(\frac{i_1 - b_1}{a_1 m}; k, \alpha_1\right)$$

2. Protein expression after cytosolic cargo release by single-lipoplexes:

$$f_{I_1,I_2}(i_1, i_2) = \sum_{k=0}^{+\infty} \mathrm{NegBino}(k; \kappa, \theta) \sum_{x=0}^{k} \mathrm{Bino}(x; k, p_e)\mathbf{1}\left(\frac{i_2 - b_2}{a_2} = mx - \frac{i_1 - b_1}{a_1}\right)\text{Bino}\left(\frac{i_1 - b_1}{a_1 m}; x, \alpha_1\right)$$

3. Cell-associated cargo delivered by mingle-lipoplexes:

$$f_{I_1,I_2}(i_1,i_2) = \sum_{k=0}^{+\infty} \mathrm{NegBino}(k;\kappa,\theta)\mathbf{1}\left(\frac{i_2-b_2}{a_2} = mk - \frac{i_1-b_1}{a_1}\right)\mathrm{Bino}\left(\frac{i_1-b_1}{a_1};km,\alpha_1\right)$$

4. Protein expression after cytosolic cargo release by mingle-lipoplexes:

$$f_{I_1,I_2}(i_1,i_2)$$
$$= \sum_{k=0}^{+\infty} \mathrm{NegBino}(k;\kappa,\theta) \sum_{x=0}^{k} \mathrm{Bino}(x;k,p_e)\mathbf{1}\left(\frac{i_2-b_2}{a_2}\right.$$
$$\left. = mx - \frac{i_1-b_1}{a_1}\right)\mathrm{Bino}\left(\frac{i_1-b_1}{a_1};mx,\alpha_1\right)$$

Supplementary Fig. 11 shows examples of these joint distributions $f_{I_1,I_2}(i_1,i_2)$ and their corresponding marginal distributions $f_{I_j}(i_j)$. The parameters used were $\alpha_1 = \alpha_2 = 0.5, m = 5$ (i.e., 5 cargo molecules per lipoplex), $\kappa = 2$, $\theta = 5$ (so that the average number of cell-associated lipoplexes is $\kappa\theta = 10$), $p_e = 0.3$ (i.e., 30% probability that a cell-associated lipoplex releases its cargo into the cytosol). In addition, we used $a_1 = a_2 = 1$ and $b_1 = b_2 = 0$, as other values would only result in a scaling and offset of the distribution, respectively. The discrete nature of the distributions in essence comes from the assumption of having a discrete number of cargo molecules per lipoplex. In case of single-lipoplexes this means that the number of cargo molecules of each type always are present as a multiple of $m = 5$. In case of mingle-lipoplexes it is rather the sum of both types of cargo molecules that always is a multiple of $m = 5$. Accounting for a variable number of cargo molecules per lipoplex and a variable number of labels/expressed proteins per cargo molecule might provide for a more accurate description. Also, including experimental noise into the models would

make the distributions continuous which would better match the experimental flow cytometry plots in the main manuscript. While in future work it may be of interest to introduce these factors into the model to make it more complete, it is important to note that those factors do not influence the changes in correlation between single and mingle-lipoplexes. Indeed, the current simplified model is sufficient to capture the essence of how the correlation changes between conditions, which is the primary focus of the current manuscript. For instance, the plots in Supplementary Figure 11 show how the correlation is improved when delivery happens by mingle instead of single-lipoplexes (compare B with A and D with C). Also the loss in correlation upon cargo release and protein expression, is visually clear (compare C with A and D with B) since the distributions become less elongated, or more spherical so to say.

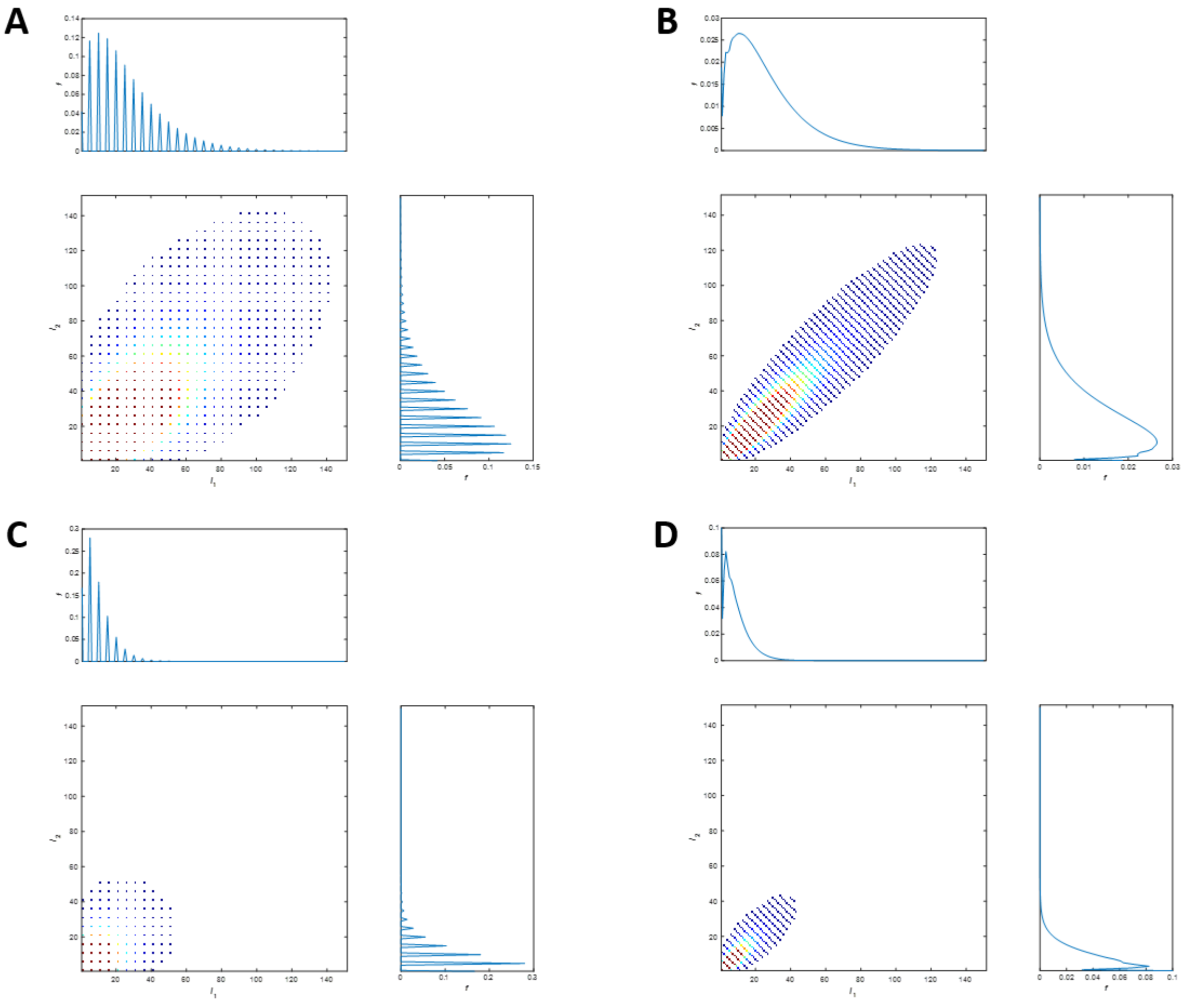

Supplementary Figure 11. Exemplary joint distributions $f_{I_1,I_2}(i_1, i_2)$ and marginal distributions $f_{I_j}(i_j)$ for (A) cell-associated cargo molecules delivered by single-lipoplexes, (B) cell-associated cargo molecules delivered by mingle-lipoplexes, (C) fluorescent protein expression by single-lipoplexes, and (D) fluorescent protein expression by mingle-lipoplexes. Parameters used were: $\alpha_1 = \alpha_2 = 0.5$, $m = 5$, $\kappa = 2$, $\theta = 5$, $p_e = 0.3$, $a_1 = a_2 = 1$ and $b_1 = b_2 = 0$.

These observations remain the same, when the actual fitting parameters $\mathrm{D}(K) = 3.62, p_e = 0.14$ and $m = 27.0$, as derived from the model, were used. As explained above, in order to generate plots of the full distribution model an assumption has to be made on the distribution of K. As argued above, a Negative Binomial distribution is a reasonable assumption: $f_K(k) = \mathrm{NegBino}(k; \kappa, \theta)$. In that case, the coefficient of dispersion is $D(K) = 1 + \theta$, which fixes parameter $\theta = 2.62$. Parameter κ can be determined from the average number of lipoplexes per cell: $\mathrm{E}[K] = \kappa\theta$. However, this is unknown in the present study so that we have to make a reasonable assumption for this. Assuming 10 lipoplexes on average per cell (similar to how Suppl. Fig. 11 was created), we have that $\kappa = \frac{10}{2.62} = 3.82$. In addition, we used $a_1 = a_2 = 1$ and $b_1 = b_2 = 0$, as other values would only result in a scaling and offset of the distribution, respectively. For this set of parameters extra plots were created for the case $\alpha_1 = \alpha_2 = 0.5$, as shown in Supplementary Figure 12.

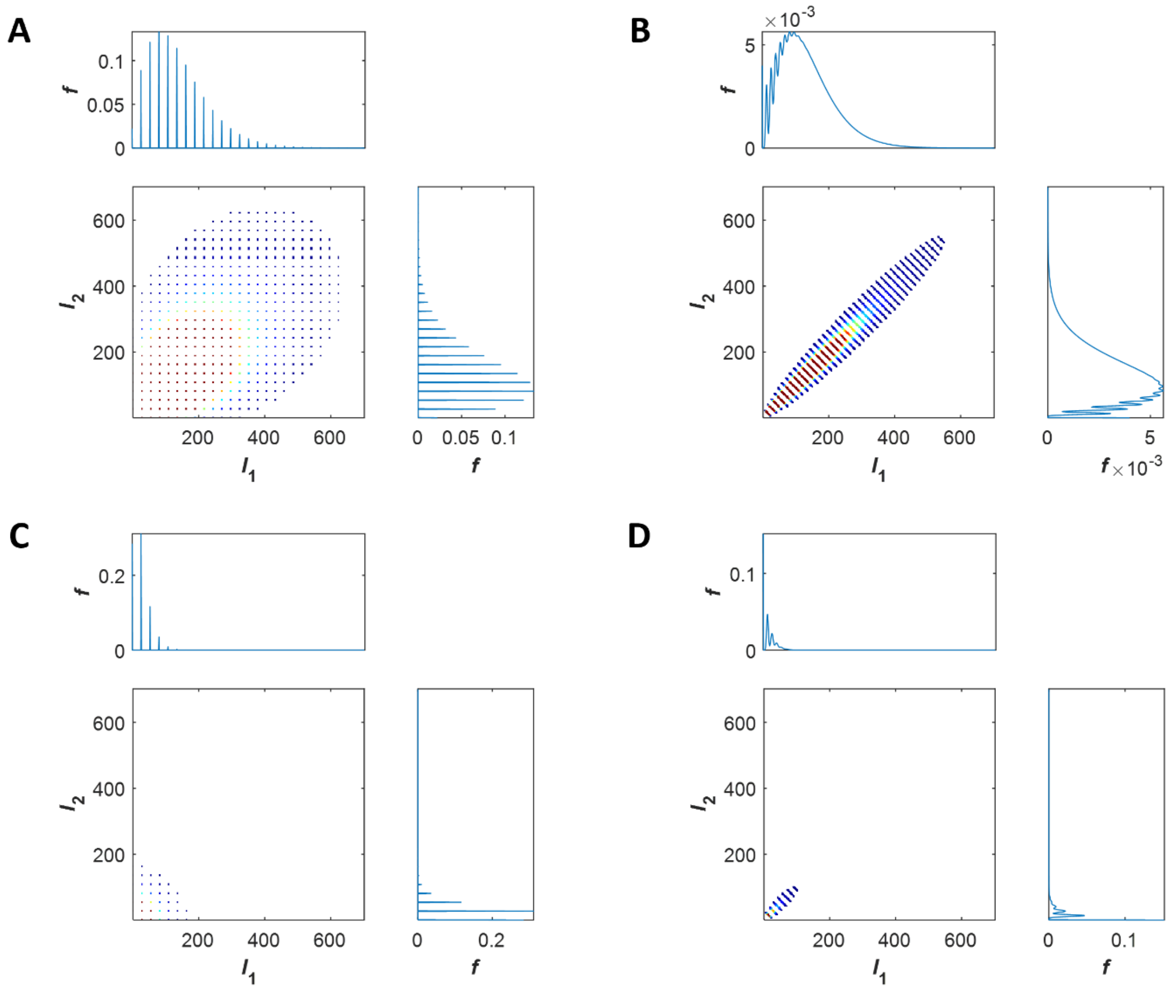

Supplementary Figure 12. Joint distributions $f_{I_1,I_2}(i_1, i_2)$ and marginal distributions $f_{I_j}(i_j)$ for (A) cell-associated cargo molecules delivered by single-lipoplexes, (B) cell-associated cargo molecules delivered by mingle-lipoplexes, (C) fluorescent protein expression by single-lipoplexes, and (D) fluorescent protein expression by mingle-lipoplexes. Parameters used were: $\alpha_1 = \alpha_2 = 0.5$, $m = 27$, $\kappa = 3.82$, $\theta = 2.62$, $p_e = 0.14$, $a_1 = a_2 = 1$ and $b_1 = b_2 = 0$.

6. Correlation analysis

In the present manuscript we are mostly concerned with understanding the correlation between uptake and expression from two types of mRNAs delivered by single- and mingle-lipoplexes. As already discussed above and in the main text, the theory derived here does qualitatively predict the observed trends in correlation between experiments. In addition, it is of interest to see to which

extent the theoretical model can describe the experimental correlation values in a quantitative way. For convenience and clarity we here repeat the relevant expressions:

1. Cell-associated cargo delivered by single-lipoplexes:

$$\text{Corr}(I_1, I_2) = \text{Corr}(L_1, L_2) = \frac{\mathrm{D}(K) - 1}{\sqrt{\left(1 + \rho_\alpha \mathrm{D}(K)\right)(1 + \mathrm{D}(K)/\rho_\alpha)}}$$

2. Protein expression after cytosolic cargo release by single-lipoplexes:

$$\text{Corr}(I_1, I_2) = \text{Corr}(N_1, N_2) = \frac{\mathrm{D}(X) - 1}{\sqrt{(1 + \mathrm{D}(X)\rho_\alpha)(1 + \mathrm{D}(X)/\rho_\alpha)}}$$

3. Cell-associated cargo delivered by mingle-lipoplexes:

$$\text{Corr}(I_1, I_2) = \text{Corr}(L_1, L_2) = \frac{mD(K) - 1}{\sqrt{\left(1 + m\rho_\alpha D(K)\right)(1 + m\,D(K)/\rho_\alpha)}}$$

4. Protein expression after cytosolic cargo release by mingle-lipoplexes:

$$\text{Corr}(I_1, I_2) = \text{Corr}(N_1, N_2) = \frac{mD(X) - 1}{\sqrt{\left(1 + m\rho_\alpha D(X)\right)(1 + m\,D(X)/\rho_\alpha)}}$$

where

$$\rho_\alpha = \frac{\alpha_1}{\alpha_2} = \frac{\alpha_1}{1 - \alpha_1}$$

$$\mathrm{D}(K) = \frac{\mathrm{Var}(K)}{\mathrm{E}[K]}$$

$$\mathrm{D}(X) = \frac{\mathrm{Var}(X)}{\mathrm{E}[X]} = 1 + p_e(D(K) - 1)$$

Since $\mathrm{D}(X)$ can be expressed as a function of $\mathrm{D}(K)$, in effect those 4 expressions are completely determined by the parameters $\mathrm{D}(K)$, p_e, m and ρ_α. $\mathrm{D}(K)$ is related to the probability distribution of the number of lipoplexes associated with cells. If all experiments corresponding to those 4 cases are performed under identical conditions (i.e., same cell type and density, lipoplex composition, lipoplex concentration, incubation time etc.), then $\mathrm{D}(K)$ should be the same for all. Similarly, if lipoplexes are prepared under identical conditions for single- and mingle- lipoplexes, then also m should be the same for all experiments. The same goes for p_e when considering transfection experiments. Therefore, since ρ_α is known *a priori* for each experiment, the different experimental correlation values that all belong to the same incubation time can be fitted all at once by the above four expressions with $\mathrm{D}(K)$, p_e and m as global fitting parameters. If only three parameters can quantitatively describe the correlation values from our diverse sets of experiments, it is a clear indication that the model is a good description of reality. This turns out to be the case, as discussed in the main manuscript.

Appendix 1: **Covariance and correlation of uptake of single-lipoplexes**

To calculate the covariance, we use the law of total covariance,

$$\mathrm{Cov}(K_1, K_2) = \underbrace{\mathrm{E}[\mathrm{Cov}(K_1, K_2|K)]}_{\mathrm{I}} + \underbrace{\mathrm{Cov}(\mathrm{E}[K_1|K], \mathrm{E}[K_2|K])}_{\mathrm{II}}$$

Starting with the term I, the covariance is

$$\mathrm{Cov}(K_1, K_2|K) = \mathrm{E}[K_1 K_2|K] - \mathrm{E}[K_1|K]\mathrm{E}[K_2|K].$$

Since $f_{K_i|K}(k_i; k) = \mathrm{Bino}(k_i; k, \alpha_i)$ we have that $E[K_i|K = k] = k\alpha_i$ and $\mathrm{Var}(K_i|K = k) = k\alpha_i(1 - \alpha_i) = k\alpha_1\alpha_2$. Combined with using that $K = K_1 + K_2$ we obtain that for $K = k$ that

$$\begin{aligned}
\mathrm{Cov}(K_1, K_2|K=k) &= E[K_1(K-K_1)|K=k] - k\alpha_1 k\alpha_2 \\
&= E[K_1 K|K=k] - E[K_1^2|K=k] - k^2\alpha_1\alpha_2 \\
&= kE[K_1|K=k] - (\mathrm{Var}(K_1|K=k) + \mathrm{E}[K_1|K=k]^2) - k^2\alpha_1\alpha_2 \\
&= k^2\alpha_1 - (k\alpha_1\alpha_2 + (k\alpha_1)^2) - k^2\alpha_1\alpha_2 = k^2\alpha_1(1-\alpha_1) - k\alpha_1\alpha_2 - k^2\alpha_1\alpha_2 \\
&= k^2\alpha_1\alpha_2 - k\alpha_1\alpha_2 - k^2\alpha_1\alpha_2 \qquad\qquad = -k\alpha_1\alpha_2
\end{aligned}$$

For term I we finally obtain that

$$E[\mathrm{Cov}(K_1, K_2|K)] = E[-K\alpha_1\alpha_2] = -\alpha_1\alpha_2 E[K].$$

For term II we find that

$$\mathrm{Cov}(\mathrm{E}[K_1|K], \mathrm{E}[K_2|K]) = \mathrm{Cov}(K\alpha_1, K\alpha_2) = \alpha_1\alpha_2 \mathrm{cov}(K, K) = \alpha_1\alpha_2 \mathrm{Var}(K)$$

so that finally we obtain for the covariance

$$\mathrm{Cov}(K_1, K_2) = -\alpha_1\alpha_2 E[K] + \alpha_1\alpha_2 \mathrm{Var}(K) = \alpha_1\alpha_2(\mathrm{Var}(K) - E[K])$$

from which the correlation immediately follows as

$$\begin{aligned}
\mathrm{Corr}(K_1, K_2) &= \frac{\mathrm{Cov}(K_1, K_2)}{\sqrt{\mathrm{Var}(K_1)\mathrm{Var}(K_2)}} = \frac{\alpha_1\alpha_2(\mathrm{Var}(K) - E[K])}{\sqrt{\left(\alpha_1\alpha_2\mathrm{E}[K] + \alpha_1^2\mathrm{Var}(K)\right)\left(\alpha_1\alpha_2\mathrm{E}[K] + \alpha_2^2\mathrm{Var}(K)\right)}} \\
&= \frac{\mathrm{D}(K) - 1}{\sqrt{\left(1 + \rho_\alpha \mathrm{D}(K)\right)(1 + \mathrm{D}(K)/\rho_\alpha)}}
\end{aligned}$$

where we have made use of the expression for $\mathrm{Var}(K_i)$ already derived in the main text and defined the ratio $\rho_\alpha = \frac{\alpha_1}{\alpha_2}$ and $\mathrm{D}(K) = \frac{\mathrm{Var}(K)}{\mathrm{E}(K)}$ as the coefficient of dispersion of K.

Appendix 2: Covariance and correlation of uptake of mingle-lipoplexes

Completely analogous to Appendix 1, the correlation can again be calculated starting from the law of total covariance:

$$\mathrm{Cov}(L_1, L_2) = \underbrace{\mathrm{E}[\mathrm{Cov}(L_1, L_2|K)]}_{\mathrm{I}} + \underbrace{\mathrm{Cov}(E[L_1|K], \mathrm{E}[L_2|K])}_{\mathrm{II}}$$

Starting with term I, we first calculate the covariance:

$$\text{Cov}(L_1, L_2|K) = \text{E}[L_1 L_2|K] - \text{E}[L_1|K]\text{E}[L_2|K]$$

We already determined before that $\text{E}[L_i|K = k] = k\alpha_i m$, so that we only need to look at the first term:

$$\text{E}[L_1 L_2|K = k] = E[L_1(mK - L_1)|K = k] = mE[L_1 K|K = k] - E[L_1^2|K = k]$$

$$= mkE[L_1|K = k] - (\text{Var}(L_1|K = k) + \text{E}[L_1|K = k]^2)$$

$$= \alpha_1 (mk)^2 - (\alpha_1 mk(1 - \alpha_1) + (\alpha_1 mk)^2)$$

So that we find:

$$\text{Cov}(L_1, L_2|K = k) = \alpha_1 (mk)^2 - (\alpha_1 mk\alpha_2 + (\alpha_1 mk)^2) - \alpha_1 \alpha_2 (mk)^2$$

$$= \alpha_1 (mk)^2 (1 - \alpha_1) - \alpha_1 mk\alpha_2 - \alpha_1 \alpha_2 (mk)^2$$

$$= \alpha_1 (mk)^2 \alpha_2 - \alpha_1 \alpha_2 mk - \alpha_1 \alpha_2 (mk)^2 = -\alpha_1 \alpha_2 mk$$

The first term I then becomes

$$\text{E}[\text{Cov}(L_1, L_2|K)] = -\alpha_1 \alpha_2 m \text{E}[K]$$

For term II we find

$$\text{Cov}(E[L_1|K], \text{E}[L_2|K]) = \text{Cov}(\alpha_1 mK, \alpha_2 mK) = \alpha_1 \alpha_2 m^2 \text{Cov}(K, K) = \alpha_1 \alpha_2 m^2 \text{Var}(K)$$

Putting this all together we find for the covariance:

$$\text{Cov}(L_1, L_2) = -\alpha_1 \alpha_2 m \text{E}[K] + \alpha_1 \alpha_2 m^2 \text{Var}(K) = \alpha_1 \alpha_2 m(m\text{Var}(K) - \text{E}[K])$$

The correlation then is:

$$\text{Corr}(L_1, L_2) = \frac{\alpha_1\alpha_2 m(m\text{Var}(K) - E[K])}{\sqrt{\big(\alpha_1\alpha_2 mE[K] + (\alpha_1 m)^2\text{Var}(K)\big)\big(\alpha_1\alpha_2 mE[K] + (\alpha_2 m)^2\text{Var}(K)\big)}}$$

$$= \frac{\alpha_1\alpha_2\left(m\frac{\text{Var}(K)}{E[K]} - 1\right)}{\sqrt{\alpha_1\alpha_2\left(1 + \frac{\alpha_1}{\alpha_2}m\frac{\text{Var}(K)}{E[K]}\right)\alpha_2\alpha_1\left(1 + \frac{\alpha_2}{\alpha_1}m\frac{\text{Var}(K)}{E[K]}\right)}}$$

$$= \frac{mD(K) - 1}{\sqrt{\big(1 + m\rho_\alpha D(K)\big)(1 + m\,D(K)/\rho_\alpha)}}$$

where as before we defined the coefficient of dispersion $D(K) = \frac{\text{Var}(K)}{E[K]}$ and the ratio $\rho_\alpha = \frac{\alpha_1}{\alpha_2}$.
